# Pan-KRAS Inhibitors BI-2493 and BI-2865 Display Potent Antitumor Activity in Tumors with KRAS Wild-type Allele Amplification

**DOI:** 10.1158/1535-7163.MCT-24-0386

**Published:** 2024-12-21

**Authors:** Antonio Tedeschi, Fiorella Schischlik, Francesca Rocchetti, Johannes Popow, Florian Ebner, Daniel Gerlach, Antonia Geyer, Valeria Santoro, Andrew S. Boghossian, Matthew G. Rees, Melissa M. Ronan, Jennifer A. Roth, Jesse Lipp, Matthias Samwer, Michael Gmachl, Norbert Kraut, Mark Pearson, Dorothea Rudolph

**Affiliations:** 1 Boehringer Ingelheim RCV GmbH & Co KG, Vienna, Austria.; 2 Broad Institute of MIT and Harvard, Cambridge, Massachusetts.

## Abstract

KRAS^G12C^ selective inhibitors, such as sotorasib and adagrasib, have raised hopes of targeting other *KRAS*-mutant alleles in patients with cancer. We report that *KRAS* wild-type (WT)–amplified tumor models are sensitive to treatment with the small-molecule KRAS inhibitors BI-2493 and BI-2865. These pan-KRAS inhibitors directly target the “OFF” state of KRAS and result in potent antitumor activity in preclinical models of cancers driven by KRAS-mutant proteins. In this study, we used the high-throughput cellular viability Profiling Relative Inhibition Simultaneously in Mixtures assay to assess the antiproliferative activity of BI-2493 in a 900+ cancer cell line panel, expanding on our previous work. *KRAS* WT–amplified cancer cell lines, with a copy number >7, were identified as the most sensitive, across cell lines with any *KRAS* alterations, to our pan-KRAS inhibitors. Importantly, our data suggest that a KRAS “OFF” inhibitor is better suited to treat *KRAS* WT–amplified tumors than a KRAS “ON” inhibitor. *KRAS* WT amplification is common in patients with gastroesophageal cancers in which it has been shown to act as a unique cancer driver with little overlap to other actionable mutations. The pan*-*KRAS inhibitors BI-2493 and BI-2865 show potent antitumor activity *in vitro* and *in vivo* in *KRAS* WT–amplified cell lines from this and other tumor types. In conclusion, this is the first study to demonstrate that direct pharmacologic inhibition of KRAS shows antitumor activity in preclinical models of cancer with *KRAS* WT amplification, suggesting a novel therapeutic concept for patients with cancers bearing this *KRAS* alteration.

## Introduction

Activating *KRAS* alterations occur in approximately one in seven of all human cancers, making it one of the most prevalent oncogenic drivers (1, 2). Gain-of-function missense mutations in *KRAS* are particularly frequent in pancreatic and colorectal cancer and lung adenocarcinoma (2, 3). Focal, high-level amplification of the *KRAS* wild-type (WT) allele, an event highly prevalent in gastroesophageal cancer, can additionally result in KRAS activation (4). Although inhibitors that address the KRAS^G12C^ oncoprotein have received clinical approval, effective therapies targeting other *KRAS* alterations, including *KRAS* WT amplification, remain to be identified.

KRAS is a small GTPase acting as a molecular switch driving intracellular proliferation and survival pathways (5, 6). *KRAS* mutations result in the accumulation of active (GTP-bound, “ON”) KRAS and persistent pathogenic activation of downstream signaling (7). The majority of *KRAS* mutations compromise, but do not entirely eliminate, the intrinsic hydrolytic activity of KRAS. Therefore, similar to WT KRAS, mutated KRAS cycles between the active and inactive (GDP-bound, “OFF”) states (8–10). It has therefore been possible to develop inhibitors that address and lock KRAS^G12C^ in the “OFF” state, preventing its return to a GTP-bound “ON” state (8, 11–14). Accordingly, sotorasib and adagrasib have received accelerated approval because of the clinical benefit observed in pretreated patients with advanced or metastatic non–small cell lung cancer (NSCLC) harboring *KRAS*^G12C^ mutations (15, 16).

Evidence that KRAS can be effectively addressed has driven intense research efforts and clinical entry of inhibitors selectively addressing other *KRAS* mutant alleles and pan-(K)RAS inhibitors that can target a broad spectrum of KRAS-mutant proteins (2). The development and clinical entry of the RAS^MULTI^ inhibitor RMC-6236 by Revolution Medicines is an elegant example of the latter approach (17, 18). Early signs of clinical benefit have been observed in patients receiving RMC-6236 (18, 19), which targets all three isoforms of the RAS family (HRAS*,* NRAS, and KRAS). Although preliminary safety data are encouraging, its long-term tolerability remains to be evaluated. We have previously reported on the identification of BI-2493 and BI-2865 (10), two compounds with a pan-KRAS inhibition profile. These pan-KRAS inhibitors target KRAS in the inactive “OFF” state, while sparing HRAS and NRAS, and exert antitumor activity in preclinical cancer models (10). The structurally related KRAS^multi^ inhibitor BI 3706674 has recently entered clinical trials in patients with solid tumor with *KRAS*^G12V^ mutations or WT *KRAS* amplification [ClinicalTrials.gov Identifier: NCT06056024 (20, 21)]. Selectively targeting KRAS has the potential for improved tolerability because of compensation of KRAS inhibition by other RAS family members in normal tissues. Preclinical models have demonstrated that *KRAS* is essential for embryonic development and postpartum and that sustained *RAS* signaling is required for skin homeostasis (22–25).

*KRAS* WT–amplified cancers represent a considerable (∼7%) fraction of *KRAS*-driven cancers, with an incidence of 9,000 patients every year in the United States, based on data across seven major cancer types (2). This alteration is particularly common in stomach and esophageal cancers (2), which are the fifth and seventh most common causes of cancer mortality worldwide, respectively, and together account for more than 1.1 million deaths worldwide in 2022 (https://gco.iarc.fr/today/en/fact-sheets-cancers). Patients with unresectable or metastatic gastroesophageal adenocarcinoma or squamous cell carcinoma (SCC) have few treatment options (26). Although *KRAS* WT amplification was originally identified over 40 years ago as an oncogenic driver, it remains less characterized than the well-known canonical *KRAS* mutations (27). *KRAS* WT amplification in gastric cancers augments the level of KRAS WT “ON” and increases downstream oncogenic signaling (28). Recent work has further strengthened the concept of *KRAS* WT amplification as an oncogenic driver in gastroesophageal cancers (4, 29). In the absence of mutations, KRAS WT activation remains dependent on the activity of upstream modulators, such as the guanine nucleotide exchange factor SOS1, or the phosphatase SHP2. In agreement with this, antiproliferative activity was observed in *KRAS* WT–amplified gastric cancer models treated with inhibitors of SOS1 or SHP2 (4).

Here, we use the pan-KRAS inhibitors BI-2865 and BI-2493 to show an antiproliferative response in a range of WT *KRAS–*amplified [copy number (CN) > 7] preclinical cancer models, confirming that amplification represents a *KRAS* oncogenic driver alteration. We propose that owing to its retained hydrolytic activity and dependency on upstream activators, *KRAS* WT–amplified cancers remain sensitive to KRAS “OFF” inhibitors. These findings support the clinical development of the KRAS^multi^ inhibitor BI 3706674 in gastroesophageal cancers and other cancer types carrying *KRAS* WT amplifications.

## Materials and Methods

### Chemical compounds

BI-2493 and BI-2865 used in this study were synthesized as described in the Supplementary Material (Synthetic Methods) of reference 10.

### Profiling Relative Inhibition Simultaneously in Mixtures high-throughput screen, data processing, and integration

We collaborated with the Broad Institute of MIT and Harvard to perform a high-throughput multiplexed cancer cell line viability screening, here referred to as Profiling Relative Inhibition Simultaneously in Mixtures (PRISM) screen. BI-2493 was screened in the PRISM assay using an eight-point dose approach (in threefold dilutions) with a 5-day treatment. The minimum and maximum doses were chosen at 0.00457 and 10 µmol/L, respectively. A total of 801 cancer cell lines passed quality control. Two distinct cell line collections were used for screening: PR500 (only adherent cell lines) and PR300+ (adherent and suspension cell lines). Detailed description of the protocol, including barcoding, culturing conditions, treatment, and controls, are extensively explained in the PRISM publication (30) and on the PRISM website (https://www.theprismlab.org/, accessed on March 10, 2022).

Response vectors are given as IC_50_ or AUC values (AUC is equivalent to the “activity area”) for each cell line. The AUC value is the area derived under the dose–response curve and ranges from 0 to 1. Higher values indicate drug resistance, which is why drug sensitivity is given as 1 − AUC. From a technical point of view, an AUC value larger than 1 is feasible (and would indicate cells proliferating more with drug treatment); however, it is also associated with noise, as higher variability is seen with concentrations that show lack of inhibition. Therefore, AUC values are capped at 1 for PRISM data. For the analysis described in this report, we use either IC_50_ or AUC values. If a direct comparison of potency across compounds is required, IC_50_ values are preferred. Otherwise, we use AUC as AUC provides quantitative values for a larger proportion of cell lines tested (IC_50_ is undefined for resistant cell lines, in which viability does not decrease below 50%). The sensitivity threshold of 0.15 (with values above 0.15 considered sensitive) was defined based on the median sensitivity (1 − AUC) across all *KRAS* WT cell lines (cell lines without *KRAS* amplification or mutations in the *KRAS* gene).

The source code for PRISM data processing, including curve fit procedures, is available on GitHub (https://github.com/cmap/dockerized_mts, RRID: SCR_002630).

### Integration of BI-2493 sensitivity data with molecular features of cancer cell lines

The cancer cell lines used in PRISM screens were molecularly characterized through the Cancer Cell Line Encyclopedia (CCLE; ref. 31) and are made publicly available through the DepMap portal (https://depmap.org/portal/, accessed on June 06, 2023, DepMap Public version 22Q4). For the analysis of this study, we focused on gene mutations (*OmicsSomaticMutations.csv*), expression(*OmicsExpressionProteinCodingGenesTPMLogp1.csv*), and CN alterations (*OmicsCNGene.csv*). In this study, for CN alterations, unless otherwise specified, gene-level relative CN data were log_2_-transformed with a pseudocount of 1, log_2_ (CN ratio + 1). The CN ratio is a relative CN estimate and more specifically, relative to the ploidy of the cell line. Cell lines with an amplified WT *KRAS* gene (without mutations in *KRAS*) were grouped into cell lines with a relative CN of >2 or >7 of the *KRAS* gene.

Data from Project Achilles (https://depmap.org/portal/achilles/, accessed on March 10, 2022) were used to estimate tumor gene dependencies (also referred to as gene essentialities), using high-throughput genome-scale CRISPR loss-of-function screens. The exact method is described in the original publication (32). For this study, we used Chronos scores (bioRxiv 10.1101/720243) from Project Achilles combined with scores from Project Score (33) from the Wellcome Sanger Institute. The scores were scaled so that the median score of the common essentials across all cell lines is −1.0, which is a commonly used threshold for calling a gene “dependent” or “essential.” A score equal or close to 0 indicates genes that are not essential. Gene dependency scores are publicly available through the DepMap portal (https://depmap.org/portal/, accessed on March 10, 2022, DepMap Public version 22Q2, *CRISPRGeneEffect.csv*). For RNAi dependency data, we used a combined set of three large-scale datasets: the Broad Institute Project Achilles, Novartis Project DRIVE, and the Marcotte and colleagues (34) breast cell line data. Cancer cell line dependencies were estimated using demeter2 (https://depmap.org/R2-D2/; ref. 35). Gene dependency scores for RNAi data are publicly available through the DepMap portal (https://depmap.org/portal/, accessed on March 10, 2022, *D2_combined_gene_dep_scores.csv*). The terms gene dependency and gene effect have the same meaning in this study.

We collected signature scores as surrogate markers of *RAS* transcriptional activity. The MAPK pathway activity score (MPAS) was collected from Wagle and colleagues (36). Briefly, for each sample, the MPAS activity score was derived from expression data for 10 MAPK pathway–specific genes (*PHLDA1*, *SPRY2*, *SPRY4*, *DUSP4*, *DUSP6*, *CCND1*, *EPHA2*, *EPHA4*, *ETV4*, and *ETV5*). The remaining signatures were collected from (37). Signature scores were estimates from gene expression data using single-sample gene set enrichment using the R package *GSVA* (RRID: SCR_021058). Signature gene annotations were mapped to the RNA-seq feature IDs for CCLE cell lines.

Data from the drug screens (valid AUC values for 801 cell lines) and genomic features were matched using their unique cell line identifier (CCLE name). A total of 795 cell lines had data for *KRAS* mutation status (*KRAS* amplification and *KRAS* point mutations), 795 cell lines had expression data, 795 cell lines had CN information, 622 cell lines had CRISPR data, and 458 had RNAi gene dependency data available.

### The Cancer Genome Atlas data

For this study, we focused on two large cohorts of multiomic datasets. The Cancer Genome Atlas (TCGA) is composed of 11,000 samples in 33 tumor types. Relative CN data were used to query for *KRAS* amplifications. Mutation data were used to exclude samples with mutations in the *KRAS* gene.

For survival analysis, we used a stratified Cox regression while controlling for confounding factors of gender and ethnicity using the R package *survival* (RRID: SCR_021137). Kaplan–Maier curves were generated using the R package *survminer* (RRID: SCR_021094).

### American Association for Cancer Research GENIE data

We selected patients from the GENIE cohort (v15.1-public; ref. 38) and focused our analysis on a subset of samples derived from Memorial Sloan Kettering, the largest American Association for Cancer Research (AACR) GENIE cohort. We further filtered for alterations listed as “somatic” and “profiled in all queried genes/profiles.” After filtering, the subcohort was composed of 70,407 patients. *KRAS*-amplified samples were defined as having a genomic identification of significant targets in cancer (GISTIC), version 2.0 score of 2 according to AACR GENIE. Exact CN thresholds are not available from the AACR GENIE cohort.

We selected esophagogastric cancers from Memorial Sloan Kettering and Dana-Farber Cancer Institute. These encompass the following cancer types: esophageal adenocarcinoma, stomach adenocarcinoma, adenocarcinoma of the gastroesophageal junction, esophagogastric adenocarcinoma, and esophageal SCC.

### Cell culture

Tumor cell lines were obtained from ATCC, the Korean Cell Line Bank (KCLB), the RIKEN Cell Bank, the Japanese Cancer Research Resources Bank (JCRB) or the German Collection of Microorganisms and Cell Culture (Deutsche Sammlung von Mikroorganismen und Zellkulturen). All cell lines used in this study were cultured for less than eight passages after master bank thawing according to the manufacturer’s instructions. All master bank aliquots were routinely authenticated by short tandem repeat (STR, conducted at Eurofins Genomics) analysis and tested for the absence of *Mycoplasma* infection (MycoAlert assay, Lonza Bioscience, or Mycoplasma Real-Time PCR Applied Biosystems #4384772) at Boehringer Ingelheim. Source and testing dates for master bank aliquots of used cell lines were as follows: HEK293T (ATCC CRL-1573, RRID: CVCL_0045, MycoAlert August 21, 2020, STR profile September 29, 2020), A-375 (ATCC CRL-1619, RRID: CVCL_0132, MycoAlert July 11, 2023, STR profile August 16, 2023), NCI-H520 (ATCC HTB-182, RRID: CVCL_1566, MycoAlert April 14, 2021, STR profile April 21, 2021), SNU-1079 (KCLB 01079, RRID: CVCL_5008, MycoAlert September 01, 2022, STR profile November 22, 2022), SNU-478 (KCLB 00478, RRID: CVCL_5065, MycoAlert September 01, 2022, STR profile September 21, 2022), NCI-H838 (ATCC CRL-5844, RRID: CVCL_159, MycoAlert May 22, 2019, STR profile October 15, 2019), NCI-H661 (ATCC HTB-183, RRID: CVCL_1577, MycoAlert January 22, 2018, STR profile February 27, 1018), HSKT-C (RIKEN RCB0515, RRID: CVCL_8234, MycoAlert November 03, 2022, STR profile November 22, 2022), SAS (JCRB JCRB0260, RRID: CVCL_1675, MycoAlert August 18, 2022, STR profile November 22, 2022), SNU-245 (KCLB 00245, RRID: CVCL_5038, MycoAlert September 01, 2022, STR profile September 21, 2022), HuG1-N (RIKEN RCB1179, RRID: CVCL_4846, MycoAlert November 03, 2022, STR profile November 22, 2022), BEN (Deutsche Sammlung von Mikroorganismen und Zellkulturen ACC 254, RRID: CVCL_1082, MycoAlert September 22, 2022, STR profile November 22, 2022), SNU-1196 (KCLB 01196, RRID: CVCL_5015, MycoAlert October 18, 2022, STR profile December 16, 2022), SNU-685 (KCLB 00685, RRID: CVCL_5083, MycoAlert September 22, 2022, STR profile November 22, 2022), Kuramochi (JCRB JCRB0098, RRID:CVCL_1345, MycoAlert October 20, 2021, STR profile December 28, 2021), MKN1 (JCRB JCRB0252, RRID: CVCL_1415, MycoAlert September 22, 2022, STR profile December 16, 2022), UMC-11 (ATCC CRL-5975, RRID: CVCL_1784, MycoAlert November 27, 2020, STR profile November 25, 2020), KE-39 (RIKEN RCB1434, RRID: CVCL_3385, MycoAlert September 27, 2022, STR profile November 22, 2022), KLE (ATCC CRL-1622, RRID: CVCL_1329, Mycoplasma Real-Time PCR December 20, 2013, STR profile January 15, 2013), and DMS 53 (ATCC CRL-2062, RRID: CVCL_1177, MycoAlert May 16, 2018, STR profile July 10, 2018).

For *in vitro* proliferation assays, cells were seeded at 500 cells per well in 40 μL of growth medium in a white bottom opaque 384-well plate and allowed to grow overnight. To obtain starting densities, a set of cells seeded in parallel was lysed and measured using the CellTiter-Glo luminescent cell viability reagent (Promega product code G7570) per well as per the manufacturer’s recommendation. The compounds were added to the cells at logarithmic dose series using an HP Digital Dispenser D300 (Tecan), normalizing for added DMSO. After compound addition, the cells were incubated for 5 days, and the viability was measured using the CellTiter-Glo reagent as described above.

To determine DUSP6 transcript modulation *in vitro*, cells were seeded at 10,000 cells in 200 µL culture medium per well in a 96-well plate. Cells were allowed to adhere overnight. Compound dilutions were added from DMSO stock solutions using an HP D300 Dispenser (Tecan; final DMSO concentration, 0.1%). Typically, cells were incubated at 37°C for 2 hours. Treated cells were processed using the FastLane Cell Multiplex Kit (Qiagen product code 216513). Threshold cycle numbers were determined using an AriaMx Real-time PCR System (Agilent Technologies) and GAPDH (Applied Biosystems Cat. #4326317E-1301046, VIC) and DUSP6 (Life Technologies Cat. #Hs00169257_m1, FAM) probes. Results were analyzed by relative quantification and are specified as the percentage of matched DMSO controls.

For pERK *in vitro* inhibition assays, 50,000 cells were seeded per well in 96-well plates in 200 µL growth medium. Cells were allowed to adhere overnight. Compound dilutions were added from DMSO stock solutions using an HP D300 Dispenser (Tecan; final DMSO concentration, 0.1%). Cells were incubated with compounds as indicated, the medium was removed, and the cells were washed with 50 µL ice-cold PBS. Cells were then lysed in 50 µL of lysis buffer (Meso Scale Discovery product code R60TX-2) supplemented with 1:100 protease inhibitor solution, 1:100 phosphatase inhibitor I, and 1:100 phosphatase inhibitor II (Meso Scale Discovery product code R70AA-1) added to each well. Plates with lysis buffer were immediately transferred to −80°C freezer for at least 2 hours and then thawed on ice. Lysates were analyzed using the Phospho (Thr202/Tyr204; Thr185/Tyr187)/Total ERK1/2 assay whole-cell lysate kit (Meso Scale Discovery product code K15107D-3) as recommended by the manufacturer.

For *in vitro* analysis of cell cycle states, cells were seeded in triplicate in a 96-well flat-bottom tissue culture plate followed by resting overnight. Compounds were added in indicated concentrations with an HP Digital Dispenser D300 (Tecan) normalized for added DMSO. After 48 hours of growth, cells were analyzed with the Click-iT EdU Alexa Fluor 647 Flow Cytometry Assay Kit (Thermo Fisher Scientific, #C10419) according to the manufacturer’s protocol with slight modifications to account for the 96-well format. In brief, cells were incubated with 10 µmol/L 5-ethynyl-2′-deoxyuridine (EdU) for 2 hours, harvested with TrypLE Express (Gibco, # 12604039) followed by 4% paraformaldehyde fixation and saponin-based permeabilization. EdU detection was realized by a click reaction of an azide coupled to Alexa Fluor 647 to the alkyne found in EdU. After the click reaction, DNA was additionally stained with FxCycle Violet Stain (Thermo Fisher Scientific, #F10347). Fluorescent signals were measured by flow cytometry on CytoFLEX LX (Beckman Coulter) and analyzed using FlowJo V10.10 (BD, RRID: SCR_008520).

For flow cytometry–based analysis of apoptosis, cells were seeded in triplicate in a 96-well flat-bottom tissue culture plate followed by resting overnight. Compounds were added in indicated concentrations with an HP Digital Dispenser D300 (Tecan) normalized for added DMSO. For the positive control of apoptosis induction, camptothecin (from Apoptosis Inducer Set, Merck, #APT800) was added in the indicated concentration. After 24 and 48 hours, cells were harvested with TrypLE Express (Gibco, #12604039), followed by incubation of cells at 37°C for 60 minutes in media containing 0.1 nmol/L MitoStatus TMRE (BD, #564696), a dye that accumulates within healthy, intact mitochondria. Thereafter, cells were pelleted and resuspended in 100 µL Annexin V Binding Buffer (BioLegend, #422201) containing 5 µL Annexin V APC (BioLegend, #640941) per well followed by incubation at room temperature for 15 minutes. After pelleting and resuspending in Annexin V Binding Buffer, cells were measured on CytoFLEX LX (Beckman Coulter) and analyzed using FlowJo V10.10 (BD).

### Biomarker and pharmacokinetic/pharmacodynamic analysis

pERK modulation in tumors was determined using the Phospho/Total ERK1/2 whole-cell lysate kit (Meso Scale Diagnostics, K15107D) as recommended by the manufacturer. Briefly, tumors were homogenized in lysis buffer (Meso Scale Discovery product code R60TX-2) supplemented with 1:100 phosphatase inhibitor cocktail #2 (Sigma product code P5726), phosphatase inhibitor cocktail #3 (Sigma product code P0044), protease inhibitor cocktail (Roche product code 11836170001), 1 mmol/L phenylmethanesulfonyl fluoride (Sigma product code P7626), and 0.1% SDS (Sigma product code 75746) using ReadyPrep mini grinders (Bio-Rad product code 163-2146), ReadyPrep mini grinders, and resin tubes (Bio-Rad product code 163-2146) as recommended by the manufacturer. Lysates were cleared by centrifugation (10 minutes, 10,000 × *g*) and analyzed using a MESO SECTOR S 600 reader (Meso Scale Discovery).

For DUSP6 modulation in tumors, RNA was isolated using the RNeasy mini kit (Qiagen product code 73404). Less than 50 mg tumor (typically around 10 mg) was homogenized in 900 µL QIAzol lysis reagent using a TissueLyser II device (Qiagen, 1 minute, 30 Hz), adding a 5-mm stainless steel bead per tube. All further steps of RNA purification were carried out as specified by the manufacturer using 1-bromo-2-chloropropane (Sigma product code B9673) instead of chloroform. RNA samples were analyzed using the QuantiGene singleplex assay kit (Invitrogen product code QS0013) and singleplex RNA probes (Invitrogen product code QGS-200, assay IDs SA-11958 for DUSP6 and SA-10057 for *TBP* as a houskeeping gene).

### Mouse studies

For cell line–derived xenograft (CDX) models, mice were housed according to the internal institutional and Austrian governmental and European Union guidelines (Austrian Animal Protection Laws, ETS-123) at Boehringer Ingelheim. All animal studies were approved by the internal ethics and the local governmental committee. To establish CDX xenograft models, 7- to 8-week-old female NMRI nude mice from Taconic (BomTac:NMRI-*Foxn1nu*, RRID: IMSR_TAC:NMRINU) were engrafted subcutaneously with 5 million cells (DMS 53 or MKN1), suspended in 50% growth factor–reduced, phenol red–free Matrigel (Corning). Mice were group-housed under pathogen-free and controlled environmental conditions (21 ± 1.5°C temperature, 55% ± 10% humidity, and a 12 hour light–dark cycle). For efficacy studies, once tumors reached roughly 200 mm³ volume, mice were randomized based on tumor size (*n* = 7 mice per treatment arm) and treated with either vehicle control [0.5% Natrosol/5% 2-hydroxylpropyl-β-cyclodextrin (HPβCD)] or BI-2493 at 30 or 90 mg per kg twice daily. Treatment was administered by oral gavage using an application volume of 10 mL per kg. The average tumor diameter (two perpendicular axes of the tumor were measured) was measured in control and treated groups using a caliper in a nonblinded manner by a research technician, who was not aware of the objectives of the study. Tumor volumes were measured 2 to 3 times a week, whereas animals were weighed and examined daily for clinical signs. Animals were sacrificed based on severity criteria (humane endpoints), including body weight, tumor size (a maximum tumor size of 1,500 mm³ was approved by the ethics committee), and tumor necrosis. For pharmacodynamic studies, once MKN1 tumors reached roughly 200 to 400 mm³ volume, mice were randomized based on tumor size (*n* = 5 mice per treatment arm) and treated with either vehicle control (0.5% Natrosol/5% HPβCD) or BI-2493 at 90 mg per kg twice daily for three consecutive days. Plasma and tumors were collected 6 or 24 hours after the penultimate treatment.

For patient-derived xenograft (PDX) models, studies were conducted at Crown Bioscience, China. Four- to five-week-old female NOD/SCID (NOD/ShiLtJGpt-Prkdcem26Cd52/Gpt-Ncr1/Gpt, RRID: IMSR_GPT:T054080) mice were implanted with tumor fragments (2–3 mm in diameter) into the right flank via trocar implant. When the tumor volume reached approximately 200 mm³, mice were randomized based on tumor size (*n* = 8 mice per treatment arm) and treated with either vehicle control (0.5% Natrosol/5% HPβCD) or BI-2493 at 30 mg per kg twice daily. All studies were conducted following an approved institutional animal care and use committee protocol. During the study, the care and use of animals were conducted in accordance with the regulations of the Association for Assessment and Accreditation of Laboratory Animal Care. Animal experiments were approved by the institutional animal care and use committees of Boehringer Ingelheim Regional Center Vienna GmbH & Co KG or Crown Bioscience Inc. Tumor volumes were measured 2 to 3 times weekly, whereas animals were weighed and examined daily and sacrificed based on severity criteria (humane endpoints), including body weight, tumors size (a maximum tumor size of 1,500 mm³ was approved by Boehringer Ingelheim’ Ethics Committee), and tumor necrosis. CN for the PDX models is absolute CN derived from whole-exome sequencing using the *CopyWriteR* package algorithm (RRID: SCR_025864).

### Histopathology and IHC

IHC was performed on formalin-fixed, paraffin-embedded tissue. We used a rabbit anti-Ki67 antibody (Cell Signaling Technology, Cat. #9027, RRID: AB_2636984), diluted 1:400, for visualization of proliferative cells and a rabbit anti-cCasp3 (Cell Signaling Technology, Cat. #9664, RRID: AB_2070042), diluted 1:400, for visualization of apoptosis. Briefly, blocks were sectioned at 2 to 4 μm thickness, deparaffinized, and staining of the rehydrated sections performed in the Leica Bond RX autostainer, using citrate pH 6 HIER antigen retrieval (ER1, Leica #AR9961) and a casein blocking (CANDOR #110500)–based staining protocol. Stained slides were counterstained (EprediaGemini AS stainer), dehydrated, coverslipped, and scanned using the Leica Aperio AT2 slide scanner. Digital e-Slides were morphologically assessed, and quantitative analysis was performed using Indica Labs software HALO AI (RRID: SCR_018350).

### Statistical analysis

A correlation coefficient between two variables was assessed using the Pearson correlation (Pearson *r*). Comparisons of groups were carried out using a nonparametric two-sided Wilcoxon test. A *P* value ≤ 0.05 was considered statistically significant. For histopathology, a nonparametric unpaired Mann–Whitney test for comparison of two groups and an unpaired Kruskal–Wallis test including Dunn correction for multiple testing of three groups were used. For DUSP6 *in vitro* modulation, *P* values were calculated using two-way ANOVA, followed by the Tukey multiple comparisons test.

### Data availability

Data are provided in Supplementary Tables S1–S5. Data processing from raw intensity values to computed AUC and IC_50_ values for each dose–response curve is described in the PRISM publication (30), and the analysis code is made publicly available by the Broad Institute on GitHub (https://github.com/broadinstitute/dockerized_mts, accessed on March 10, 2022). Analysis code will be provided upon request.

## Results

### A large cancer cell line panel reveals the sensitivity of KRAS WT–amplified cell lines to pan-KRAS inhibitors

**Figure 1.**
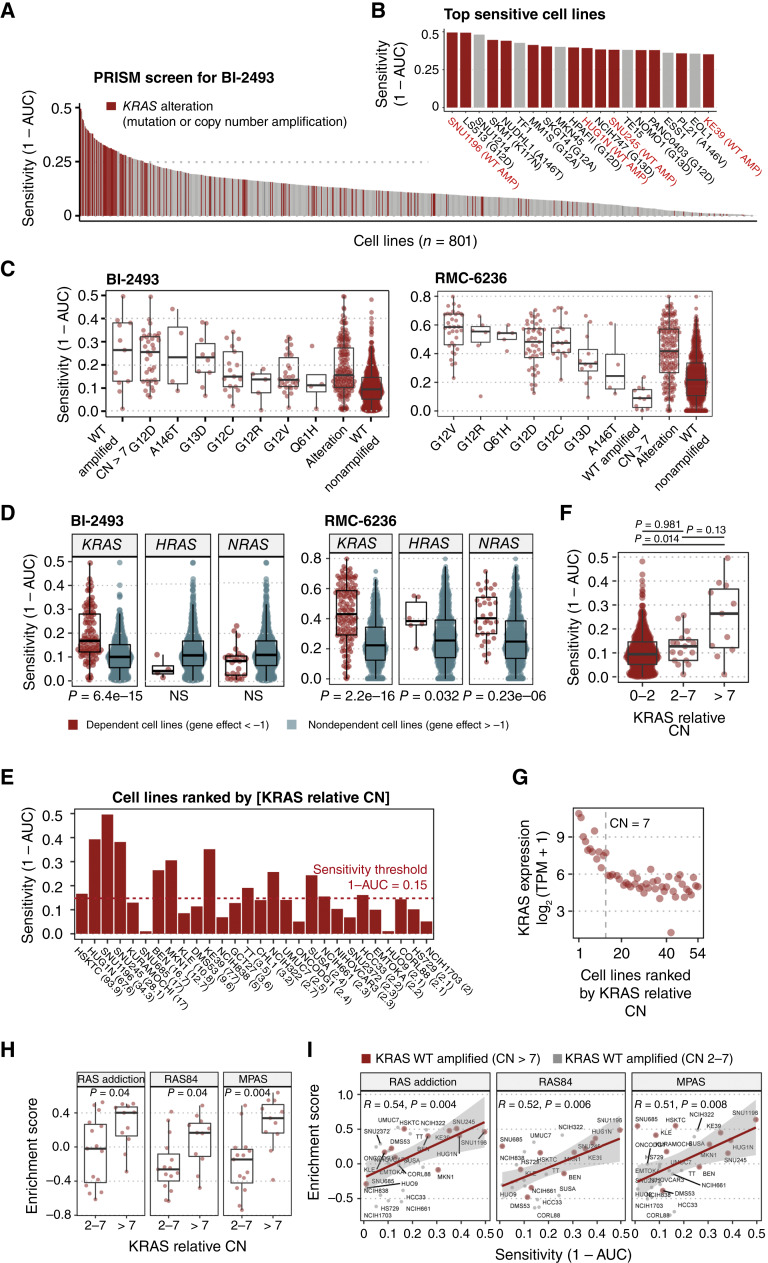
PRISM platform–based high-throughput screen with more than 900 cancer cell lines shows sensitivity of BI-2493 across a wide range of *KRAS*-altered cell lines. **A,** Sensitivity data of 801 cell lines after quality control are shown (Supplementary Table S1). Cancer cell lines are ranked from the highest (left) to lowest (right) sensitivity using the measure 1 − AUC (area under the dose–response curve). Red bars highlight *KRAS*-altered cell lines, including *KRAS* mutations (point mutations, insertions, and deletions) and *KRAS* WT CN amplifications. **B,** Topmost sensitive cell lines sorted by sensitivity to BI-2493. *KRAS* WT–amplified cell lines are indicated in red letters in the figure label. The type of *KRAS* alteration is indicated in parenthesis. **C,** Antiproliferative activity of BI-2493 and RMC-6236 (18) across different *KRAS*-mutant or *KRAS* WT–amplified cell lines. Cell lines are sorted by the median sensitivity across *KRAS* alleles. **D,** Comparison of sensitivity values for BI-2493 and RMC-6236 (8) for cell lines with dependency on either *KRAS*, *HRAS*, or *NRAS*. Cell lines with a Chronos score (gene effect score) of less than −1 were considered dependent. Sensitivity means between groups were tested for significance using a one-sided Wilcoxon test. **E,** Cell lines sorted by relative CN from the highest (left) to lowest CN (right). Only cell lines in the PRISM screen with a relative CN of larger than 2 are shown. Relative CN are indicated in brackets next to the cell line name on the *x*-axis. The sensitivity threshold is indicated with a red dotted horizontal line. **F,** Sensitivity to BI-2493 increases with a larger *KRAS* relative CN. Sensitivity means between groups were tested for significance using a one-sided Wilcoxon signed-rank test. **G, ***KRAS* expression increases with higher *KRAS* CN. Only cell lines from the PRISM screen with a relative CN higher than 2 are shown. Fifty-four cell lines with *KRAS* WT amplifications are ranked from the highest (left) to lowest (right) CN on the *x*-axis. An inflection point marking the transition to *KRAS* overexpression is indicated at a *KRAS* relative CN of 7. **H, ***RAS* oncogenic activity increases in *KRAS* WT–amplified cell lines. RAS activity was assessed using publicly available signatures for RAS activation, such as *MPAS*, *RAS84*, and *RAS* addiction. Three signatures are shown in the main figure and four signatures in Supplementary Fig. S4. A single-sample enrichment score was calculated using a single-sample gene set enrichment analysis. Only cell lines with a relative CN of >2 are shown. *P* values were estimated using a one-sided Wilcoxon test. *P* values are reported after Benjamini–Hochberg correction for multiple hypothesis testing. **I, ***RAS* activation signatures are predictive for sensitivity to BI-2493. Pearson *R* correlations are shown. Only cell lines with a relative CN >2 are depicted. Cell lines with a relative CN >7 are highlighted with red dots. AMP, amplification; NS, not significant.

Sensitivity to the pan-KRAS “OFF” inhibitor BI-2493 was assessed across a large panel of cancer cell lines, using a high-throughput PRISM platform–based cellular screen, which allows pooled screening of mixtures of 900+ cancer cell lines derived from 140 tumor types (Supplementary Table S1; ref. 30). Broad activity was observed across a wide range of *KRAS*-altered cancer cell lines (Fig. 1A–C), in agreement with previous results (10). *KRAS* alterations (mutations and WT amplifications) characterized 70% (14/20) of the most sensitive cell lines (Fig. 1B). Cellular sensitivity to BI-2493 was integrated with CRISPR and RNAi gene dependency data (32). A significant correlation was observed between cell lines genetically dependent on *KRAS* depletion and sensitivity to BI-2493 (Wilcoxon *P* value = 6.4e−15). In contrast, cell lines genetically dependent on *HRAS* or *NRAS* displayed no sensitivity to BI-2493 but, in agreement with its mechanism of action, retained sensitivity to the RAS inhibitor RMC-6236 (Fig. 1D; Supplementary Fig. S1; ref. 10).

Employing a larger cellular screen than the one previously described (10) enabled assessment of the activity of BI-2493 on additional, less frequently occurring *KRAS* alleles. Four of the 20 most sensitive cell lines bear amplifications of the *KRAS* WT allele (Fig. 1B), suggesting that pan-KRAS “OFF” inhibitors could potentially also be a therapeutic option for patients with tumors driven by this alteration. An increase in sensitivity to BI-2493 was observed for cell lines with a higher CN of *KRAS* WT (Fig. 1E), in particular in cell lines with a relative CN larger than 7 (Wilcoxon *P* value = 0.014; Fig. 1F). An inflection point that coincides with a sharp increase in *KRAS* expression starting at a CN of 7 was also observed (Fig. 1G; Supplementary Fig. S2). A relative *KRAS* copy number alteration of >7 was therefore used subsequently to define a *KRAS* WT amplification.

Seven published signatures, measuring transcriptional activity of oncogenic RAS, were used to further explore the relationship between *KRAS* WT amplification and *KRAS* oncogenic activity (36, 37). A significantly higher activity of these signatures occurred in *KRAS* WT–amplified cell lines (relative CN >7) compared with cell lines with a lower relative CN (Benjamini–Hochberg *P* value < 0.05; Fig. 1H; Supplementary Fig. S3). The MPAS signature for cell lines with amplified *KRAS* WT was equivalent to cell lines with *KRAS*-mutated cell lines, further supporting *KRAS* WT amplification as an oncogenic driver (Supplementary Fig. S3). Interestingly, the high activity observed for these signatures can explain the sensitivity of some of the *KRAS* WT (nonamplified) cell lines among the top 20 sensitive cell lines (Fig. 1B; Supplementary Table S2), including the MKN45 cell line which carries an *MET* amplification, suggesting a wider therapeutic use of the pan-KRAS inhibitors in cancers beyond those with *KRAS* WT amplifications and mutations. Moreover, we find a strong correlation between signatures for KRAS activation and sensitivity to BI-2493 across the *KRAS* WT–amplified cell panel (Fig. 1I; Supplementary Fig. S4).

The hypothesis that WT KRAS, even when amplified, would predominantly be in the KRAS “OFF” state was assessed comparing the activity of two RAS “ON” inhibitors, RMC-6236 and RMC-7977, using published cell viability data (18, 39) derived from the same large cancer cell line panel (PRISM platform) used here to test BI-2493. Cell lines bearing *KRAS*-mutant alleles in which the intrinsic hydrolytic activity is most compromised were among the most sensitive to RMC-6236 and RMC-7977 (Fig. 1C; Supplementary Fig. S5). In contrast to BI-2493, KRAS WT–amplified cell lines were among the least sensitive cell lines to RMC-6236 and RMC-7977 (Fig. 1C; Supplementary Fig. S5) in agreement with the report that *KRAS* CN alterations do not significantly affect sensitivity to RMC-6236 (18).

### *KRAS* WT amplifications are common in patients with gastroesophageal cancers

**Figure 2.**
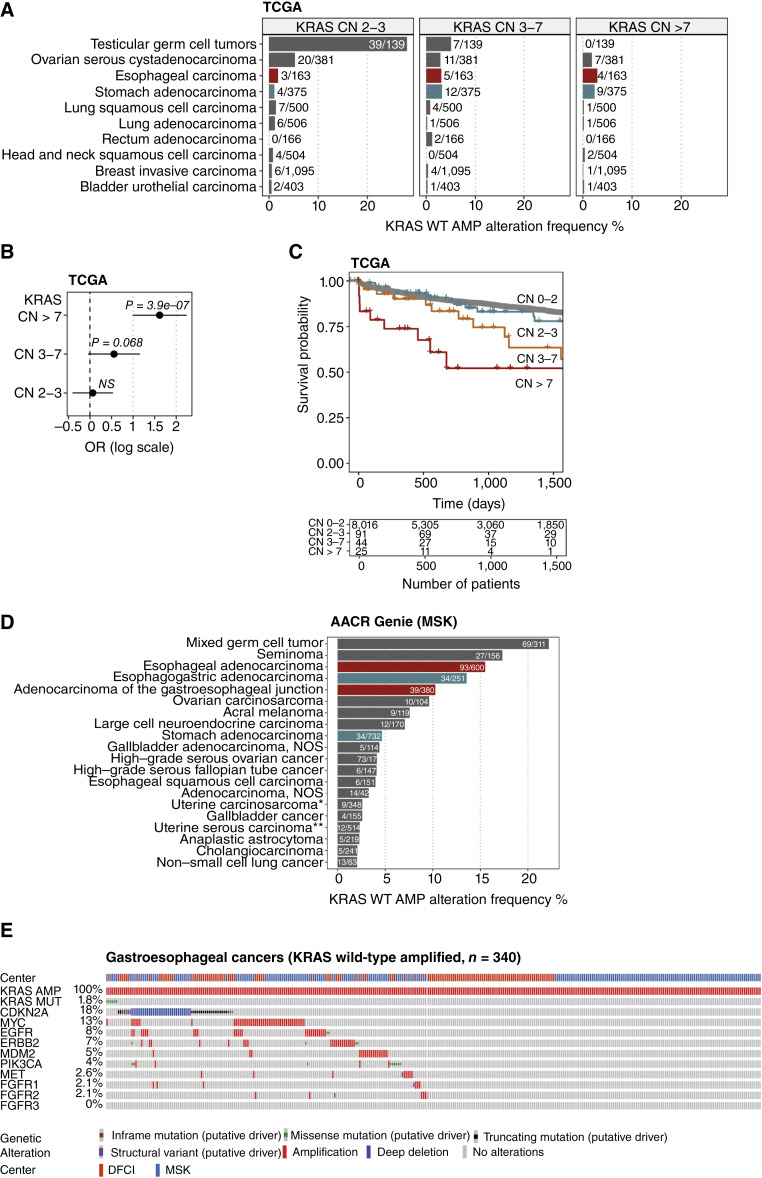
*KRAS* WT–amplified tumors are enriched in gastroesophageal cancers. **A,** TCGA cohort was queried for *KRAS* WT–amplified patients. Frequency of *KRAS* WT–amplified patients is shown, stratified by different thresholds of *KRAS* relative CNs. Only the top 10 altered cancer types are shown for CN 2 to 3. **B,** Stratified Cox regression grouped by *KRAS* CN thresholds. For each analysis, patients were grouped by a variable of interest (e.g., CN > 7) and compared with all other patients. OR plots show the OR in log scale and the lower and upper 95% confidence intervals. *P* values are indicated. **C,** Kaplan–Maier curves for TCGA patients grouped by different thresholds of *KRAS* relative CN. **D, ***KRAS* WT–amplified tumors were selected from AACR GENIE (version v15.1-public). Alterations were filtered for “somatic” and “profiled in all queried genes/profiles.” Only patients of the Memorial Sloan Kettering (MSK) cohort were selected (*n* = 70,407). The top 20 cohorts ranked by *KRAS* WT amplification frequency are shown (full cohort in Supplementary Table S3). Only cohorts with a minimum number of 100 patients are displayed. Selected counts and total counts of patients are depicted next to the bar graphs. Gastroesophageal cancers are highlighted in either blue or red. * and ** marked cancer types were abbreviated for clarity. **E,** Gastroesophageal cancers were selected from the AACR GENIE MSK and Dana-Farber Cancer Institute (DFCI) cohort (version v16.0-public). Alterations were filtered for “somatic” and “profiled in all queried genes/profiles.” Only patients with *KRAS*-amplified tumors are shown (*n* = 340); however, seven carry an additional *KRAS* mutation, reducing the number of *KRAS* WT–amplified tumors to *n* = 333. Co-alterations are ranked by frequency. *KRAS*-amplified samples are defined with a GISTIC score of 2 according to AACR GENIE. Exact CN thresholds are not available from the AACR GENIE cohort. AMP, amplification; HNSCC, SCC of the head and neck; SCLS, small cell lung cancer; NS, not significant.

The prevalence of *KRAS* WT–amplified tumors across 34 tumor types was assessed across TCGA dataset (Fig. 2A). Esophageal carcinoma (adenocarcinoma and SCC; 4/163 = 2.5%), stomach adenocarcinoma (9/375 = 2.5%), and ovarian serous cystadenocarcinoma (7/381 = 2%) showed the highest frequencies of *KRAS* WT amplification (relative CN > 7). *KRAS* WT amplification was also observed in additional tumor types, albeit at lower frequencies (Fig. 2A). As observed in cell lines, patients with a *KRAS* relative CN of >7 have a significant higher KRAS activity score based on signatures such as “MPAS,” “Ras84,” and “Ras addiction” (Supplementary Fig. S6). When patients were grouped by *KRAS* CN and a stratified Cox regression analysis was performed, patients with a CN >7 showed a highly significant, worse clinical outcome (survival) compared with patients with lower CN amplifications (Fig. 2B and C).

The frequency of *KRAS* WT–amplified tumors was analyzed across the real-world clinical genomic database AACR GENIE to confirm and expand these results. *KRAS* WT amplifications were confirmed in esophageal adenocarcinoma (∼15.5%), adenocarcinoma of the gastroesophageal junction (∼10.2%), stomach adenocarcinoma (∼4.6%), and esophageal SCC (∼4%; Fig. 2D; Supplementary Table S3). Similar frequencies were reported using an independent large real-world database from Foundation Medicine (3). The anticipated enrichment of *KRAS* WT amplifications in gastroesophageal cancers prompted an in-depth analysis of the co-mutation landscape for this tumor type (Fig. 2E; Supplementary Fig. S7). We observed that (i) *KRAS* mutations and *KRAS* WT amplifications are mutually exclusive (log_2_ OR = −1.53; *q* value < 0.001; only 7/341 patients carry both types of *KRAS* alterations) and (ii) ∼53% (180/341) of these patients carry no additional alteration in genes that encode proteins, such as ERBB2, EGFR, or MDM2, which could be otherwise pharmacologically targeted. Additionally, we found *KRAS* WT amplifications to be mutually exclusive with *ERBB2* (log_2_ OR = −1.27; *q* value < 0.001) as well as with *PIK3CA* (log_2_ OR = −1.541; *q*-value = 0.001; Supplementary Table S4). Altogether, these findings strengthen the hypothesis that *KRAS* WT amplification (CN > 7) represents an oncogenic driver in gastroesophageal cancer and identifies a patient population with a high unmet need that is likely to benefit from treatment with a WT-targeting KRAS “OFF” inhibitor.

### Pan-KRAS inhibitors block the growth of cancer cell lines with *KRAS* WT amplifications

**Figure 3.**
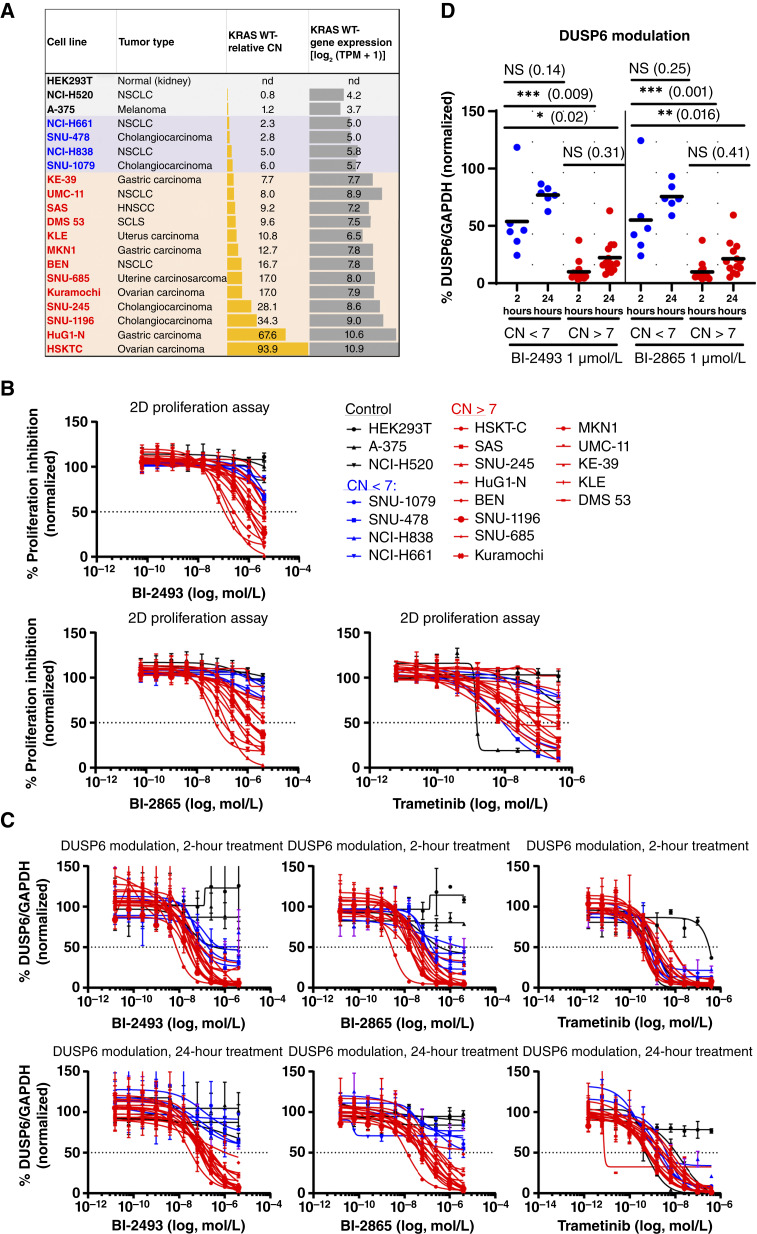
*KRAS* WT–amplified cancer cell lines are sensitive to pan-KRAS inhibitors BI-2493 and BI-2865. **A,** Cell lines tested for sensitivity to pan-KRAS inhibitors were ranked according to *KRAS* WT relative CN. Tumor of origin and *KRAS* WT gene expression [log_2_(TPM + 1)] are indicated. **B, ***In vitro* sensitivity of cell lines shown in **A** to BI-2493, BI-2865, and trametinib (*n* = 3, means ± SD). Control cell lines, cell lines with *KRAS* WT CN < 7, and cell lines with *KRAS* WT CN > 7 are colored in black, blue, and red, respectively. **C,** Downregulation of *DUSP6* mRNA by BI-2493, BI-2865, and trametinib at the indicated timepoints and for the indicated cell lines (*n* = 2, means ± SD). **D,** Quantification of downregulation of *DUSP6* mRNA from **C** at 1 µmol/L concentration of BI-2493 and BI-2865. *P* values were calculated using two-way ANOVA, followed by the Tukey multiple comparisons test. NS, not significant.

A panel of 19 cell lines, derived from different tumor types, with a range of *KRAS* WT CN amplifications was selected to further explore the antiproliferative effect of BI-2493 and BI-2865 (Fig. 3A; ref. 10). *KRAS* WT amplification correlated with *KRAS* mRNA expression across this panel of cell lines (Spearman *R* = 0.98; *P* value = 8.4e−06; Fig. 3A), in line with published data obtained from patient-derived tumor samples (4). All three *KRAS* WT–amplified gastric cancer cell lines, MKN1, HuG1-N, and KE-39, have previously been shown to have an elevated KRAS protein level and to be dependent on KRAS for proliferation (4, 28). The growth of *KRAS* WT–amplified cell lines was affected in a *KRAS* CN-dependent manner (Fig. 3B). Strikingly, BI-2493 and BI-2865 inhibited the growth of most cell lines (10/13) with KRAS CN > 7 (BI-2493 mean IC_50_ = 598 nmol/L; BI-2865 mean IC_50_ = 315 nmol/L; Supplementary Table S5). The gastric adenocarcinoma cell line HuG1-N (CN = 67) and the ovarian carcinoma cell line HSKT-C (CN = 93) were particularly sensitive to BI-2865 (IC_50_ = 30.8 nmol/L and IC_50_ = 44.7 nmol/L, respectively) and BI-2493 (IC_50_ = 59.1 nmol/L and IC_50_ = 130 nmol/L, respectively). Cell lines with a KRAS CN < 7 were not significantly affected by compound treatment (NCI-H661, SNU-478, NCI-H838, and SNU-1079; IC_50_ >4,000 nmol/L for both compounds). No effect on growth was observed in *KRAS* WT *BRAF*^V600E^ mutant A-375 melanoma cells or two *KRAS* WT, nonamplified control cell lines (HEK293T, NCI-H520, and A-375; IC_50_ >4,000 nmol/L for both compounds; Fig. 3B). These results reinforce the results of the PRISM screen that the pan-KRAS inhibitors BI-2493 and BI-2865 have potent antiproliferative activity in *KRAS *WT–amplified (CN > 7) cancer cell lines of different tumor origin.

The pharmacodynamic effects of BI-2493 and BI-2865 were further evaluated in the same cell panel. After 2 hours of drug treatment, KRAS inhibition led to downregulation of *DUSP6* mRNA and pERK levels in a dose-dependent manner, irrespective of CN, but to a higher degree in *KRAS* WT–amplified cell lines with CN > 7 (Fig. 3C and D; Supplementary Fig. S8). Notably, downregulation of the signaling pathway was maintained in *KRAS* WT–amplified CN > 7 cell lines after 24 hours of treatment (Fig. 3D; Supplementary Fig. S8B). The MEK inhibitor trametinib resulted in strong pathway modulation and an antiproliferative effect in most cell lines, irrespective of their *KRAS* WT CN status (Fig. 3; Supplementary Fig. S8). Collectively, these data show a clear correlation between the modulation of the MAPK signaling pathway and growth inhibition by pan-KRAS inhibitors in cell lines driven by *KRAS* WT amplification.

To explore the mechanism that mediates the antitumor effect of BI-2493 and BI-2865, we selected the control HEK293T cell line and the *KRAS* WT–amplified cancer cell lines SNU-245 (cholangiocarcinoma, CN = 28.1), MKN1 (gastric, CN = 12.7), and DMS 53 (NSCLC, CN = 9.6). Control HEK293T cells were not affected by treatment with pan-KRAS inhibitors, although the DNA topoisomerase I inhibitor camptothecin induces apoptosis in these cells (Supplementary Fig. S9). The pan-KRAS inhibitors induced a notable increase in apoptosis and a concomitant inhibition of proliferation in the SNU-245 cell line (Supplementary Fig. S9). However, the DMS 53 cell line only showed a modest increase in apoptosis, whereas the MKN1 cell line only showed a slight decrease in proliferating cells when treated with the pan-KRAS inhibitors for 48 hours (see values in Supplementary Fig. S9). These results are consistent with our previous report showing that the antiproliferative effect of pan-KRAS inhibitors varies in *KRAS*-mutant models (10).

### Pan-KRAS inhibitor BI-2493 suppresses tumor growth in xenograft models of *KRAS* WT–amplified cancers

**Figure 4.**
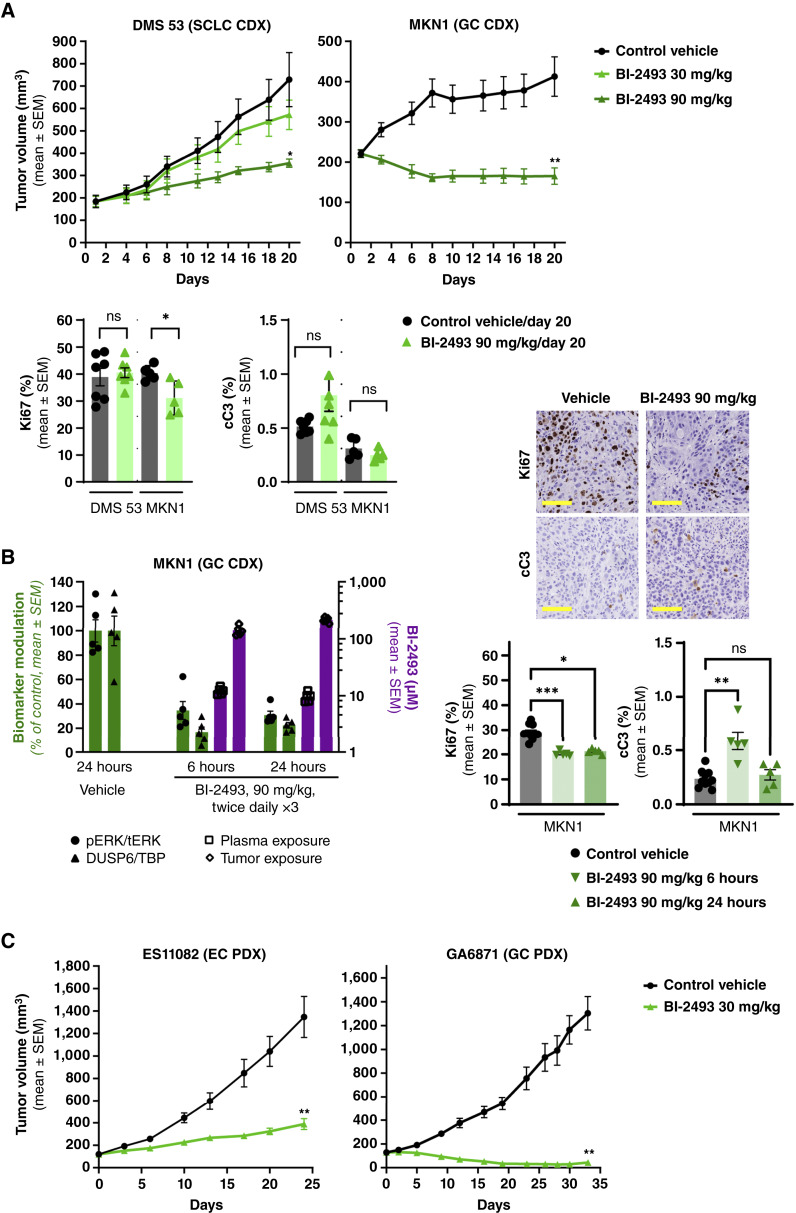
BI-2493 suppresses tumor growth in xenograft models of *KRAS* WT–amplified cancers. **A,** Antitumor activity of BI-2493 in the DMS 53 small cell lung cancer (SCLC) CDX model (left) and the MKN1 gastric cancer (GC) CDX model (right). BI-2493 was administered orally twice daily at 30 or 90 mg/kg. Data represent the mean tumor volume ± SEM (*N* = 7). Statistical significance was assessed on day 20. *, *P* < 0.05 vs. vehicle control (Wilcoxon test and Bonferroni–Holm correction). Bottom, Quantification of proliferating cells stained by Ki67 and apoptotic cells stained by cleaved caspase-3 (cC3) on tumors from the indicated models at the end of the above shown efficacy studies (day 20). Data are presented as mean ± SEM (*N* = 5). **B,** MKN1 tumor bearing mice were treated orally twice daily for three consecutive days with vehicle or with BI-2493 at 90 mg/kg. Tumors were collected 6 or 24 hours after the second last treatment and levels of ERK phosphorylation (circles) or *DUSP6* mRNA expression (triangles) were measured. BI-2493 concentrations in plasma (squares) or tumors (diamonds) at the corresponding timepoints are shown in purple. Data are presented as mean ± SEM (*N* = 5). On the right, quantification of Ki67- and cC3-positive cells from the same MKN1 samples (representative IHC images are shown). **C,** Antitumor activity of BI-2493 in the ES11082 esophageal cancer (EC) or GA6871 PDX model, respectively. BI-2493 was administered orally twice daily at 30 mg/kg. Data represent the mean tumor volume ± SEM (*N* = 8). Statistical significance was assessed on day 24 or 33, respectively. **, *P* < 0.005 vs. control vehicle (Wilcoxon test and Bonferroni–Holm correction).

BI-2493 has been optimized for *in vivo* administration and shows a favorable pharmacokinetic profile when administered orally in mice (10). A dose-dependent inhibition of tumor growth was observed upon twice daily oral administration of BI-2493 in nude mice bearing tumors derived from the *KRAS* WT–amplified small cell lung cancer cell line DMS 53 (CN = 9.5; Fig. 4A). As *KRAS* WT amplification is most frequently observed in gastric, esophageal, and gastroesophageal junction cancers, we subsequently selected CDX and PDX models from these tumor types. BI-2493 dosed orally at 90 mg/kg twice daily induced durable tumor regressions in the MKN1 *KRAS* WT–amplified (CN = 12.7, TGI = 140%) gastric cancer model (Fig. 4A). After 3 days of twice daily oral treatment with BI-2493, a strong decrease in both ERK phosphorylation and *DUSP6* mRNA expression was observed 6 hours after the second last dose and persisting until 24 hours. This is consistent with both total plasma and tumor concentrations being above the *in vitro* IC_50_ (16.4 and 81.4 nmol/L at 24 hours, respectively; Fig. 4B). We further assessed efficacy of BI-2493 in *KRAS* WT-amplified PDX models. A 30 mg/kg twice daily oral dose of BI-2493 induced tumor growth inhibition (TGI) in the esophageal cancer PDX model ES11082 (CN = 98; TGI = 78%) and led to deep and long-lasting tumor regressions in the gastric cancer PDX model GA6871 (CN = 28; TGI = 108%; Fig. 4C). BI-2493 treatment was tolerated throughout the entire duration of the studies (Supplementary Fig. S10), in agreement with our previous report (10).

We then examined the downstream consequences of KRAS inhibition in DMS 53 and MKN1 xenograft tumors at the end of the efficacy studies. In DMS 53 xenograft tumors, we did not observe either a decrease in proliferating Ki67-positive cells or an increase in apoptotic cleaved caspase-3–positive cells [Fig. 4A (bottom)], which correlates with the less pronounced antitumor effect of BI-2493 in this model. In the MKN1 xenograft tumors, which showed a regression in size upon BI-2493 treatment, we observed a significant decrease in proliferating cells [Fig. 4A (bottom)]. This decrease was confirmed on samples from MKN1 xenograft tumors collected at an earlier timepoint [Fig. 4B (right)]. In contrast, a slight increase in apoptotic cells in the MKN1 model could only be observed on samples at this early timepoint (compare cleaved caspase-3 quantification in Fig. 4B vs. 4A).

In summary, these data demonstrate that the inhibition in cellular growth *in vitro*, observed upon treatment with pan-KRAS “OFF” inhibitors, leads to suppression of tumor growth *in vivo* in preclinical models of *KRAS* WT–amplified cancers.

## Discussion

Here, we show for the first time the anticancer effects of the pan-KRAS inhibitors BI-2865 and BI-2493 in preclinical models of *KRAS* WT–amplified cancers of different tumor origin. Using these small-molecule inhibitors, we show that direct pharmacologic inhibition of KRAS in the inactive “OFF” state results in growth inhibition in *KRAS* WT–amplified cancer cell lines *in vitro* and in CDX and PDX models *in vivo*. This study brings forward a novel therapeutic concept for *KRAS* WT–amplified cancers and provides the preclinical rationale for the use of pan-KRAS inhibitors to treat patients with cancers with *KRAS* WT amplifications (CN > 7).

Pan-KRAS inhibitors BI-2865 and BI-2493 bind in a noncovalent manner to the inactive state of KRAS and prevent the activation of a broad range of mutant KRAS proteins (10). KRAS-mutant proteins sustain signaling by existing preferentially in the active “ON” state. However, the majority still cycle between the active “ON” state and the inactive “OFF” state (8, 9, 40). Previously generated biochemical data using these pan-KRAS inhibitors unveiled that KRAS-mutant proteins cycle with slower kinetics than WT KRAS (10). It is therefore conceivable that *KRAS* WT–amplified cancers will have high levels of GDP-bound WT KRAS available for binding of KRAS “OFF” inhibitors, such as BI-2493 and BI-2865. Indeed, *KRAS* WT amplification status (CN > 7) was the most significant single genetic alteration associated with response to BI-2493 in a large cancer cell line panel. Consistent with the mechanism of action, we found that *KRAS* WT–amplified cell lines show a clearly reduced sensitivity to the RAS “ON” clinical compound RMC-6236 and the tool compound RMC-7977 and they are the least sensitive cell lines among the diverse *KRAS*-mutant and *KRAS* WT–amplified cell lines that were tested in a large cancer cell line panel (18, 39). This is in line with the recent report that sensitivity to RMC-6236 is not increased in *KRAS* WT–amplified cell lines compared with nonamplified *KRAS* WT cell lines (18). Based on these data, we propose that KRAS “OFF” inhibitors are particularly well suited to treat tumors with *KRAS* WT amplifications (CN > 7).

We used the high-throughput cellular viability PRISM assay to test the antiproliferative activity of BI-2493 in a larger cancer cell line panel than in our previous work (10), which allowed us to evaluate less frequently occurring genetic alterations such as *KRAS* WT amplifications. Based on the sensitivity to BI-2493 observed in *KRAS* WT–amplified cancer cell lines, we suggest expanding the range of *KRAS* alterations that can be targeted with a pan-KRAS inhibitor and to include *KRAS* WT–amplified tumors. We confirmed these findings in proliferation assays with *KRAS* WT–amplified cell lines derived from eight different tumor types and showed KRAS pathway modulation in these cell lines, indicating a clear correlation between signaling pathway modulation and growth inhibition. Consistent with our previous report in *KRAS*-mutant cell lines (10), the antitumor response of *KRAS* WT–amplified cell lines to pan-KRAS inhibitors varies *in vitro*, and could be due, at least in some cases, to the compensatory activation of parallel pathways. We noticed that the *KRAS* WT-amplified cell lines KLE and SNU-685 are insensitive to pan-KRAS inhibitors in both the PRISM screen and CTG assays. In KLE cells, this could be due to the compensatory activation of the PI3K pathway (41). These differentiated responses are also observed *in vivo*. For example, the DMS 53 NSCLC xenograft model showed only tumor growth delay, whereas the MKN1 gastric cancer xenograft model underwent regression. We noticed that the DMS 53 model carries a missense mutation in *STK11* (*STK11* p.D194Y), which has been reported as oncogenic (42), and could explain the attenuated antitumor response to BI-2493. Overall, our *in vivo* findings corroborate the *in vitro* results, showing for the first time that direct pharmacologic inhibition of WT KRAS suppresses the growth of cancers carrying *KRAS* WT amplifications (CN > 7). Such a suppression is observed *in vitro* also upon trametinib treatment (albeit irrespective of KRAS CN). However, MEK inhibitors in the clinic have not proven as efficacious and well-tolerated as KRAS^G12C^ inhibitors (43). Similarly, we expect maximal benefit for patients with *KRAS* WT–amplified tumors to come from direct KRAS targeting through pan-KRAS inhibitors.

The sensitivity of *KRAS* WT–amplified cancer cell lines to the pan-KRAS inhibitors increases with the CN of the *KRAS* WT allele, with a concomitant increase in *KRAS* WT mRNA expression. Our data confirm these previously published results showing that *KRAS* WT amplifications are a key *KRAS* driver alteration in multiple tumor types (4, 27, 28). Furthermore, we observed that an increase in *KRAS* WT CN correlates with a higher KRAS activity score, based on the MPAS signature (36) both in cancer cell lines and in tumor samples from patients. Based on these data, we have defined a CN of 7 as the threshold to define *KRAS* WT amplifications. In agreement, based on TCGA data, patients with a *KRAS* WT CN >7 have a worse survival prognosis compared with patients with lower *KRAS* WT CN alterations. A similar observation was reported for gastric cancers (4). Here, we propose that *KRAS* WT amplifications could be used to predict sensitivity to therapy with a KRAS WT targeting pan-KRAS inhibitor. Although our preclinical data suggest a threshold for the *KRAS* WT CN of 7 that predicts sensitivity to BI-2493, retrospective analysis of tumor samples derived from patients treated with a pan-KRAS inhibitor will be required to further validate this threshold in the clinical setting.

The preclinical hypothesis presented here will require translational validation in the clinic. BI 3706674, a compound related to BI-2865 and BI-2493 with improved potency and optimized DMPK properties, has entered clinical trials in patients with gastroesophageal cancers carrying *KRAS* WT amplifications (ClinicalTrials.gov Identifier: NCT06056024). Furthermore, the broad antitumor activity across different tumor types with *KRAS* WT amplifications shown here in *in vitro* and *in vivo* preclinical models indicates the opportunity for a tumor agnostic approach of targeting *KRAS* WT–amplified tumors with our current clinical compound BI 3706674.

## Supplementary Material

Supplementary Tables 1-5 Supplementary Table 1: PRISM data integrated with omics data from depmap Supplementary Table 2:Top 20 sensitive cell lines (PRISM) Supplementary Table 3:Frequency of KRAS WT AMP in AACR Genie v 15.1 (MSK cohort) Supplementary Table 4: Mututal exclusivity for KRAS WT AMP (results downloaded from AACR Genie v15.1) Supplementary Table 5 IC50 values from CTG assays for KRAS wt amplified cell lines

Supplementary Figure 1 Supplementary Figure 1: Cellular sensitivity to BI-2493 integrated with CRISPR and RNAi gene dependency data. (Top) CRISPR: Drug-target associations show selectivity of BI-2493 for KRAS but not for HRAS and NRAS. Panel left, mid and right show gene effect (or dependency) scores derived from Chronos for KRAS, HRAS and NRAS, respectively on the x-axis. A low gene effect score indicates that a cell line is likely to depend on a given gene. A score equal to or close to 0 indicates genes that are non-essential, whereas a score of -1 is defined as the median of all common essential genes and is commonly used as a threshold for gene dependency (indicated by a red dotted vertical line). The y-axis shows drug sensitivity values reported as 1-AUC for BI-2493 derived from the PRISM screen. Larger values indicate higher sensitivity. KRAS Pearson R=-0.385, P=2.23e-23; HRAS Pearson R=0.079, P=0.0483; NRAS Pearson R=0.184, P=3.69e-06; (Bottom) RNAi: Drug-target associations show selectivity of BI-2493 for KRAS but not for HRAS and NRAS. Panel left, mid and right show gene effect (or dependency) scores derived from Demeter2 for KRAS, HRAS and NRAS, respectively on the x-axis. KRAS Pearson R=-0.393, P=2.44e-18; HRAS Pearson R=-0.0015, P=0.9; NRAS Pearson R=0.0698, P=0.14.

Supplementary Figure 2 Supplementary Figure 2: KRAS expression as a function of KRAS copy number alteration. (Left) KRAS expression increases with copy number. (Mid) High correlation of KRAS expression and copy number (Pearson R=0.904, P=4.85e-20). The vertical dotted line marks a relative copy number threshold of 7. Only cell lines with a relative copy number of >2 are shown. (Right) Ranking of relative copy number across all 800 cell lines from the PRISM screen. Vertical dotted lines from left to right mark relative copy number thresholds 10, 7, 2 and 1, respectively.

Supplementary Figure 3 Supplementary Figure 3. Relationship between KRAS wild-type amplification and KRAS oncogenic activity in cell lines. RAS activation signatures (1,3) MSigDB, KrasLA, KRASG13D134, HRAS, MPAS, ras84 and RAS addiction in KRAS wild-type amplified cell lines (relative copy number >7) compared to cell lines with a lower relative copy number (2-7) and KRAS mutated cell lines without KRAS amplification. Enrichment scores were estimated using single sample enrichment (ssGSEA). A one-sided Wilcox-test was used to test for significance between KRAS relative copy number of 2-7 or >7. Adjusted P-values (Benjamini-Hochberg) are displayed.

Supplementary Figure 4 Supplementary Figure 4. Correlation between signatures for KRAS activation and sensitivity to BI-2493 across the KRAS wild-type amplified cell panel. RAS activation signatures were obtained from East et al. (1). Correlation coefficient between enrichment scores and sensitivity to BI-2493 were estimated using a Pearson R.

Supplementary Figure 5 Supplementary Figure 5. Anti-proliferative activity of RMC-7977 across different KRAS altered cell lines. (Left) Anti-proliferative activity of RMC-7977 (2) across different KRAS mutant or KRAS wild-type amplified cell lines. Cell lines are sorted by median sensitivity across KRAS alleles. Note: AUC values are relative measures of drug sensitivity and are therefore suitable to compare drug sensitivity across cell lines for a single compound but do not allow for a comparison across compounds. (Right) Comparison of sensitivity values for RMC-7977 (2) for cell lines with dependency on either KRAS, HRAS or NRAS. Cell lines with a Chronos score (gene effect score) of less than -1 were considered dependent. Sensitivity means between groups were tested for significance using a one-sided Wilcoxon-test.

Supplementary Figure 6 Supplementary Figure 6: Relationship between KRAS wild-type amplification and KRAS oncogenic activity in TCGA patient data. RAS activation signatures MPAS (3), RAS_addiction and Ras84 (1). Enrichment scores were estimated using single sample enrichment (ssGSEA) in TCGA patient data. A one-sided Wilcox-test was used to test for significance between KRAS relative copy number of 2-7 or >7.

Supplementary Figure 7 Supplementary Figure 7. Gastroesophageal cancers are enriched in KRAS wild-typeWT amplified tumors. Gastroesophageal cancers (n=3464) were selected from the AACR Genie MSK and DFCI cohort (version v16.0-public). Only patients with any alterations in the above listed genes are shown (1498 unaltered patients are not shown). Co-alterations are ranked by frequency. KRAS amplified samples are defined with a GISTIC score of 2 according to AACR GENIE as exact copy number thresholds are not available from the AACR GENIE cohort.

Supplementary Figure 8 Supplementary Figure 8: KRAS wild-type amplified cancer cell lines are sensitive to pan-KRAS inhibitors BI-2493 and BI-2865. (A) Inhibition of pERK by BI-2493, BI-2865 and trametinib at the indicated timepoints and for the indicated cell lines (n=2, means ± SD). Control cell lines, cell lines with KRAS wild-type CN<7 and cell lines with KRAS wild-type CN>7 are colored in black, blue, and red, respectively. (B) Quantification of down regulation of pERK from (A) at 1M concentration of BI-2493 and BI-2865. P-values were calculated using two-way ANOVA, followed by Tukey’s multiple comparisons test.

Supplementary Figure 9 Supplementary Figure 9: BI-2493 and BI-2865 treatment induces cell cycle arrest and apoptosis in KRAS wild-type amplified cancer cell lines. (A) Upper panel: Representative flow blots of cell cycle states determined by EdU incorporation into newly synthesized DNA and total DNA content staining by FxCycle of indicated cell lines treated for 48 h with DMSO (mock), 3 µM BI-2493, 3 µM BI-2865 and 0.3 µM trametinib. Cells were pre-gated based on scattering properties and DNA content. Numbers indicate frequency of parent population. Lower panel: Impact of 48 h treatment with DMSO (mock), BI-2493, BI-2865 or trametinib at the indicated concentrations on cell cycle states of the indicated cell lines (N=3 (assay was run in triplicates), means + SD) (B) Upper panel: Representative flow blots of induction of apoptosis after 48 h of treatment of the indicated cell lines with DMSO (mock), 3 µM BI-2493, 3 µM BI-2865, 0.3 µM trametinib and 2 µM Camptothecin. Cells were pre-gated based on their scattering properties. Apoptotic cells were defined by loss of inner mitochondrial membrane potential (Δψm) and detection of phosphatidylserine by Annexin V staining. Numbers indicate frequency of parent population. Lower panel: Induction of apoptosis in the indicated cell lines by treatment with DMSO (mock), BI-2493, BI-2865, trametinib or Camptothecin at the indicated concentrations after 24 and 48 h determined by flow cytometry (N=3 (assay was run in triplicates), means +SD).

Supplementary Figure 10 Supplementary Figure 10. BI-2493 treatment in animal models is tolerated. % bodyweight change in xenograft models treated with control vehicle or BI-2493. Data represent the mean % bodyweight change +/- SEM of mice grafted with: (A) DMS 53 cells (N=7); (B) MKN1 cells (N=7). One animal in the BI-2493 treated group had to be sacrificed earlier (d11) due to bodyweight loss. (C) ES11082 PDX model (N=8). Two animals in the control vehicle treated group and three animals in the BI-2493 treated group had to be sacrificed earlier (d18, d18, d15, d12, d7, respectively). (D) GA6871 PDX model (N=8). One animal in the control vehicle treated group and 2 animals in the BI-2493 treated group had to be sacrificed earlier (d7 and d16, d30, respectively) due to bodyweight loss.
