# Supplementary material for: Pan-KRAS Inhibitors BI-2493 and BI-2865 Display Potent Antitumor Activity in Tumors with KRAS Wild-type Allele Amplification
Source: Mol Cancer Ther. 2024 Dec 21;24(4):550–62. doi: 10.1158/1535-7163.MCT-24-0386 (PMC11962398; doi:10.1158/1535-7163.MCT-24-0386)
Supplement: Supplementary Figure 1 — Cellular sensitivity to BI-2493 integrated with CRISPR and RNAi gene dependency data. (Top) CRISPR: Drug-target associations show selectivity of BI-2493 for KRAS but not for HRAS and NRAS. Panel left, mid and right show gene effect (or dependency) scores derived from Chronos for KRAS, HRAS and NRAS, respectively on the x-axis. A low gene effect score indicates that a cell line is likely to depend on a given gene. A score equal to or close to 0 indicates genes that are non-essential, whereas a score of -1 is defined as the median of all common essential genes and is commonly used as a threshold for gene dependency (indicated by a red dotted vertical line). The y-axis shows drug sensitivity values reported as 1-AUC for BI-2493 derived from the PRISM screen. Larger values indicate higher sensitivity. KRAS Pearson R=-0.385, P=2.23e-23; HRAS Pearson R=0.079, P=0.0483; NRAS Pearson R=0.184, P=3.69e-06; (Bottom) RNAi: Drug-target associations show selectivity of BI-2493 for KRAS but not for HRAS and NRAS. Panel left, mid and right show gene effect (or dependency) scores derived from Demeter2 for KRAS, HRAS and NRAS, respectively on the x-axis. KRAS Pearson R=-0.393, P=2.44e-18; HRAS Pearson R=-0.0015, P=0.9; NRAS Pearson R=0.0698, P=0.14. [file mct-24-0386_supplementary_figure_1_supps1.pdf]

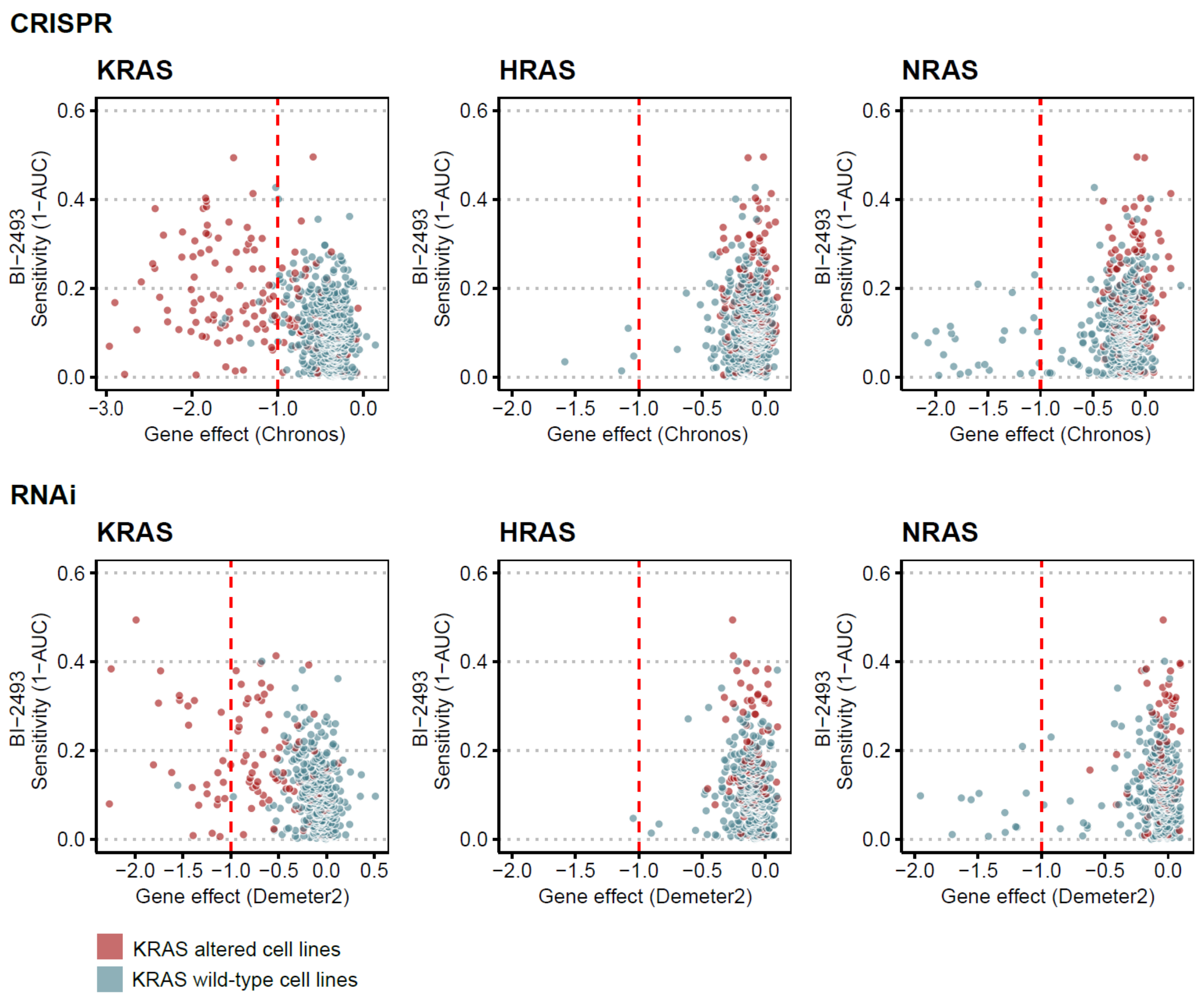

Supplementary Figure 1: *Cellular sensitivity to BI-2493 integrated with CRISPR and RNAi gene dependency data.* **(Top) CRISPR:** Drug-target associations show selectivity of BI-2493 for KRAS but not for HRAS and NRAS. Panel left, mid and right show gene effect (or dependency) scores derived from Chronos for KRAS, HRAS and NRAS, respectively on the x-axis. A low gene effect score indicates that a cell line is likely to depend on a given gene. A score equal to or close to 0 indicates genes that are non-essential, whereas a score of -1 is defined as the median of all common essential genes and is commonly used as a threshold for gene dependency (indicated by a red dotted vertical line). The y-axis shows drug sensitivity values reported as 1-AUC for BI-2493 derived from the PRISM screen. Larger values indicate higher sensitivity. KRAS Pearson R=-0.385, P=2.23e-23; HRAS Pearson R=0.079, P=0.0483; NRAS Pearson R=0.184, P=3.69e-06; (**Bottom**) **RNAi**: Drug-target associations show selectivity of BI-2493 for KRAS but not for HRAS and NRAS. Panel left, mid and right show gene effect (or dependency) scores derived from Demeter2 for KRAS, HRAS and NRAS, respectively on the x-axis. KRAS Pearson R=-0.393, P=2.44e-18; HRAS Pearson R=-0.0015, P=0.9; NRAS Pearson R=0.0698, P=0.14.
