# Supplementary material for: Pan-KRAS Inhibitors BI-2493 and BI-2865 Display Potent Antitumor Activity in Tumors with KRAS Wild-type Allele Amplification
Source: Mol Cancer Ther. 2024 Dec 21;24(4):550–62. doi: 10.1158/1535-7163.MCT-24-0386 (PMC11962398; doi:10.1158/1535-7163.MCT-24-0386)
Supplement: Supplementary Figure 2 — KRAS expression as a function of KRAS copy number alteration. (Left) KRAS expression increases with copy number. (Mid) High correlation of KRAS expression and copy number (Pearson R=0.904, P=4.85e-20). The vertical dotted line marks a relative copy number threshold of 7. Only cell lines with a relative copy number of >2 are shown. (Right) Ranking of relative copy number across all 800 cell lines from the PRISM screen. Vertical dotted lines from left to right mark relative copy number thresholds 10, 7, 2 and 1, respectively. [file mct-24-0386_supplementary_figure_2_supps2.pdf]

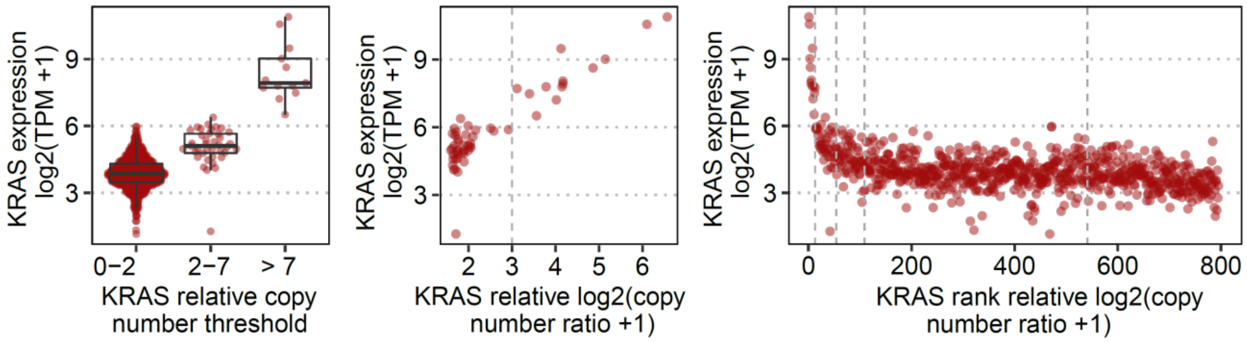

Supplementary Figure 2: *KRAS expression as a function of KRAS copy number alteration*. (**Left**) KRAS expression increases with copy number. (**Mid**) High correlation of KRAS expression and copy number (Pearson *R*=0.904, *P*=4.85e-20). The vertical dotted line marks a relative copy number threshold of 7. Only cell lines with a relative copy number of >2 are shown. (**Right**) Ranking of relative copy number across all 800 cell lines from the PRISM screen. Vertical dotted lines from left to right mark relative copy number thresholds 10, 7, 2 and 1, respectively.
