# Supplementary material for: Pan-KRAS Inhibitors BI-2493 and BI-2865 Display Potent Antitumor Activity in Tumors with KRAS Wild-type Allele Amplification
Source: Mol Cancer Ther. 2024 Dec 21;24(4):550–62. doi: 10.1158/1535-7163.MCT-24-0386 (PMC11962398; doi:10.1158/1535-7163.MCT-24-0386)
Supplement: Supplementary Figure 3 — Relationship between KRAS wild-type amplification and KRAS oncogenic activity in cell lines. RAS activation signatures (1,3) MSigDB, KrasLA, KRASG13D134, HRAS, MPAS, ras84 and RAS addiction in KRAS wild-type amplified cell lines (relative copy number >7) compared to cell lines with a lower relative copy number (2-7) and KRAS mutated cell lines without KRAS amplification. Enrichment scores were estimated using single sample enrichment (ssGSEA). A one-sided Wilcox-test was used to test for significance between KRAS relative copy number of 2-7 or >7. Adjusted P-values (Benjamini-Hochberg) are displayed. [file mct-24-0386_supplementary_figure_3_supps3.pdf]

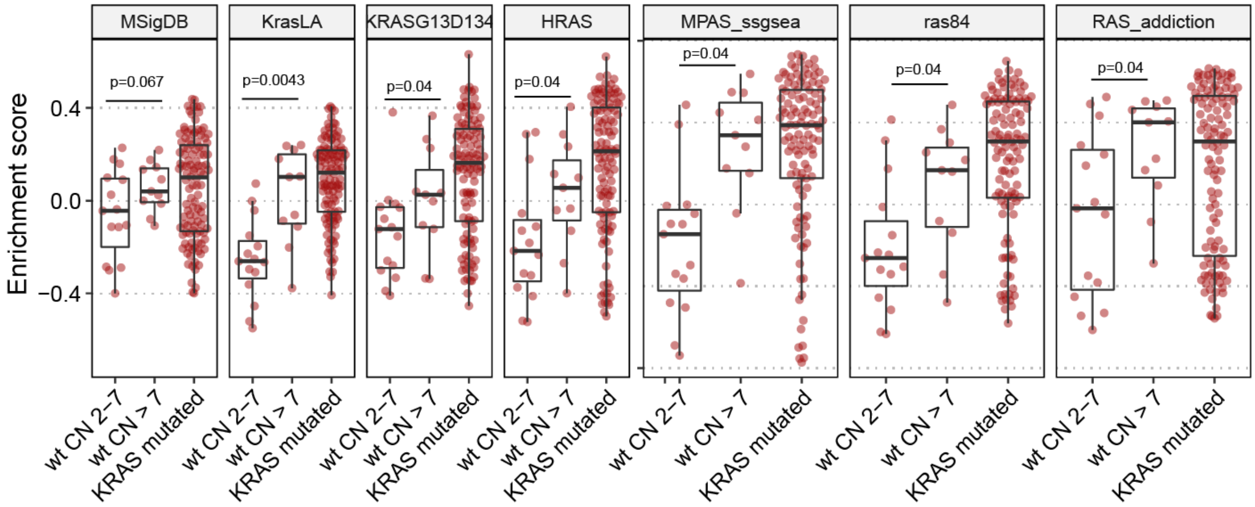

Supplementary Figure 3. *Relationship between KRAS wild-type amplification and KRAS oncogenic activity in cell lines.* RAS activation signatures (Ref. 37, 38) MSigDB, KrasLA, KRASG13D134, HRAS, MPAS, ras84 and RAS addiction in KRAS wild-type amplified cell lines (relative copy number >7) compared to cell lines with a lower relative copy number (2-7) and KRAS mutated cell lines without KRAS amplification. Enrichment scores were estimated using single sample enrichment (ssGSEA). A one-sided Wilcox-test was used to test for significance between KRAS relative copy number of 2-7 or >7. Adjusted *P-values* (Benjamini-Hochberg) are displayed.
