# Supplementary material for: Pan-KRAS Inhibitors BI-2493 and BI-2865 Display Potent Antitumor Activity in Tumors with KRAS Wild-type Allele Amplification
Source: Mol Cancer Ther. 2024 Dec 21;24(4):550–62. doi: 10.1158/1535-7163.MCT-24-0386 (PMC11962398; doi:10.1158/1535-7163.MCT-24-0386)
Supplement: Supplementary Figure 4 — Correlation between signatures for KRAS activation and sensitivity to BI-2493 across the KRAS wild-type amplified cell panel. RAS activation signatures were obtained from East et al. (1). Correlation coefficient between enrichment scores and sensitivity to BI-2493 were estimated using a Pearson R. [file mct-24-0386_supplementary_figure_4_supps4.pdf]

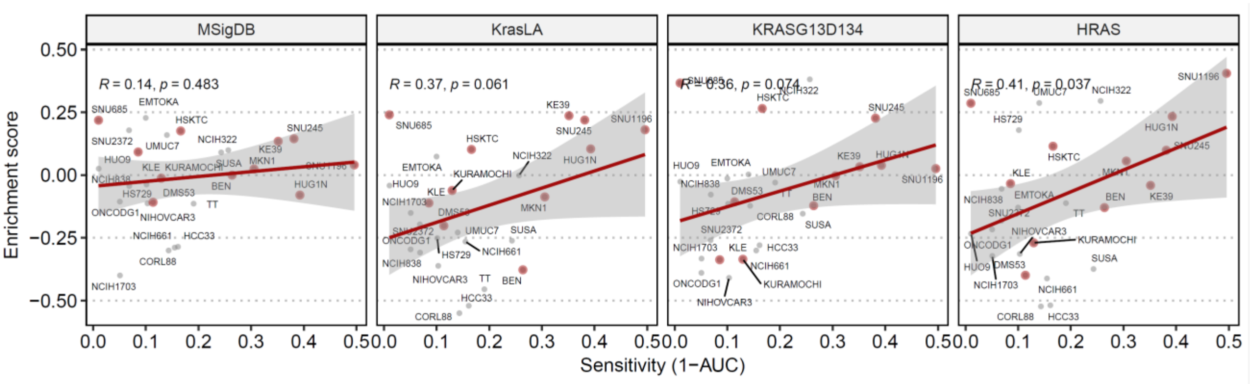

Supplementary Figure 4. *Correlation between signatures for KRAS activation and sensitivity to BI-2493 across the KRAS wild-type amplified cell panel.* RAS activation signatures were obtained from East et al. (38). Correlation coefficient between enrichment scores and sensitivity to BI-2493 were estimated using a Pearson R.

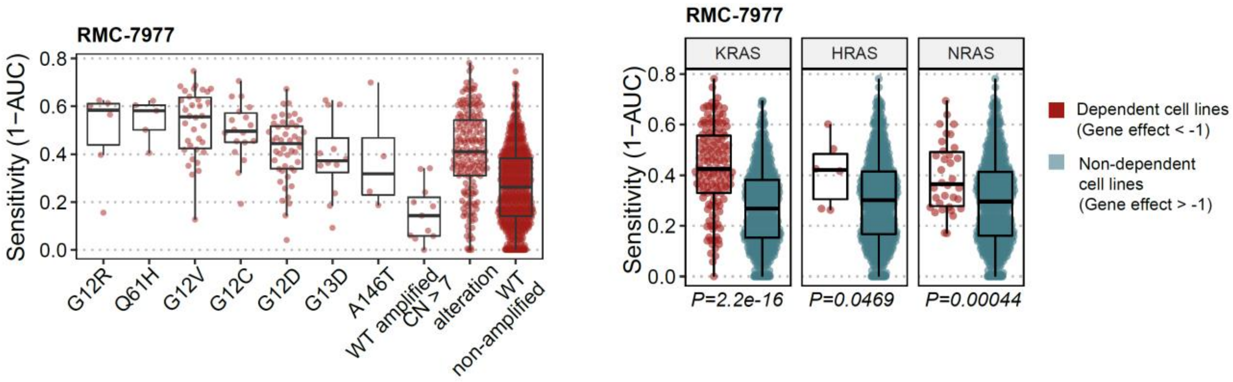

Supplementary Figure 5. *Anti-proliferative activity of RMC-7977 across different KRAS altered cell lines.* **(Left)** Anti-proliferative activity of RMC-7977 (2) across different KRAS mutant or KRAS wild-type amplified cell lines. Cell lines are sorted by median sensitivity across KRAS alleles. Note: AUC values are relative measures of drug sensitivity and are therefore suitable to compare drug sensitivity across cell lines for a single compound but do not allow for a comparison across compounds. **(Right)** Comparison of sensitivity values for RMC-7977 (2) for cell lines with dependency on either KRAS, HRAS or NRAS. Cell lines with a Chronos score (gene effect score) of less than -1 were considered dependent. Sensitivity means between groups were tested for significance using a one-sided Wilcoxon-test.

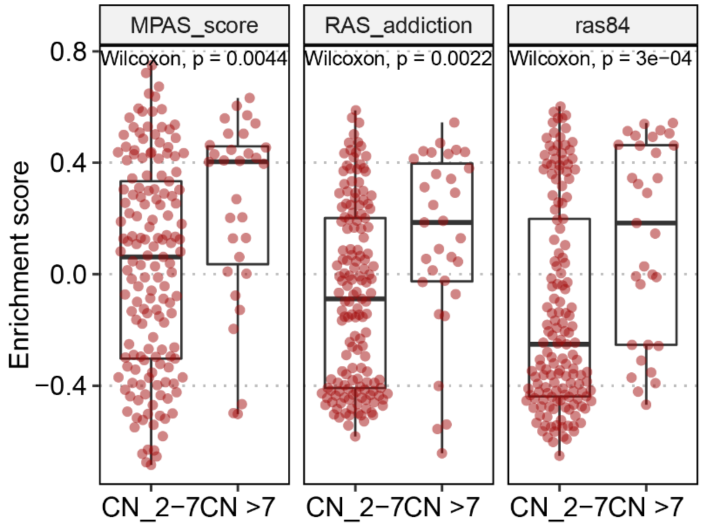

Supplementary Figure 6: *Relationship between KRAS wild-type amplification and KRAS oncogenic activity in TCGA patient data*. RAS activation signatures MPAS (3), RAS_addiction and Ras84 (1). Enrichment scores were estimated using single sample enrichment (ssGSEA) in TCGA patient data. A one-sided Wilcox-test was used to test for significance between KRAS relative copy number of 2-7 or >7.

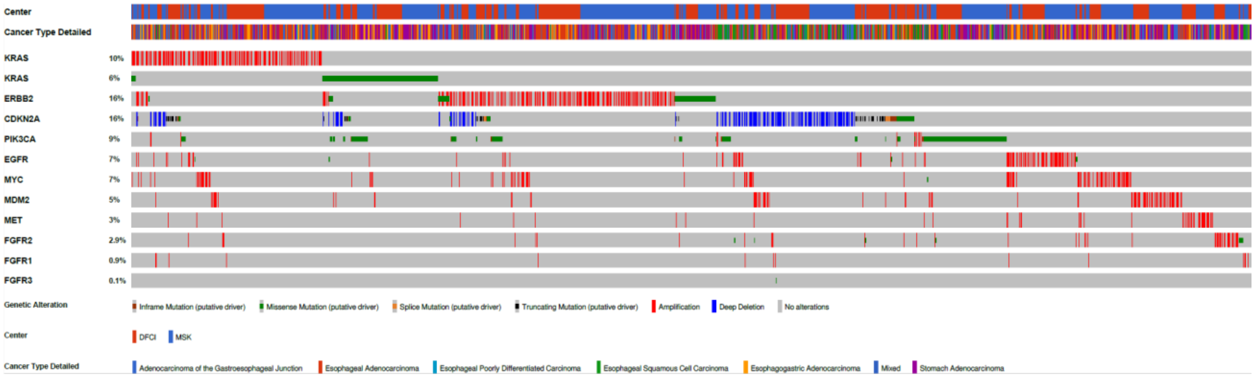

Supplementary Figure 7. *Gastroesophageal cancers are enriched in KRAS wild-type amplified tumors*. Gastroesophageal cancers *(n=3464)* were selected from the AACR Genie MSK and DFCI cohort (version v16.0-public). Only patients with any alterations in the above listed genes are shown (1498 unaltered patients are not shown). Co-alterations are ranked by frequency. KRAS amplified samples are defined with a GISTIC score of 2 according to AACR GENIE as exact copy number thresholds are not available from the AACR GENIE cohort.

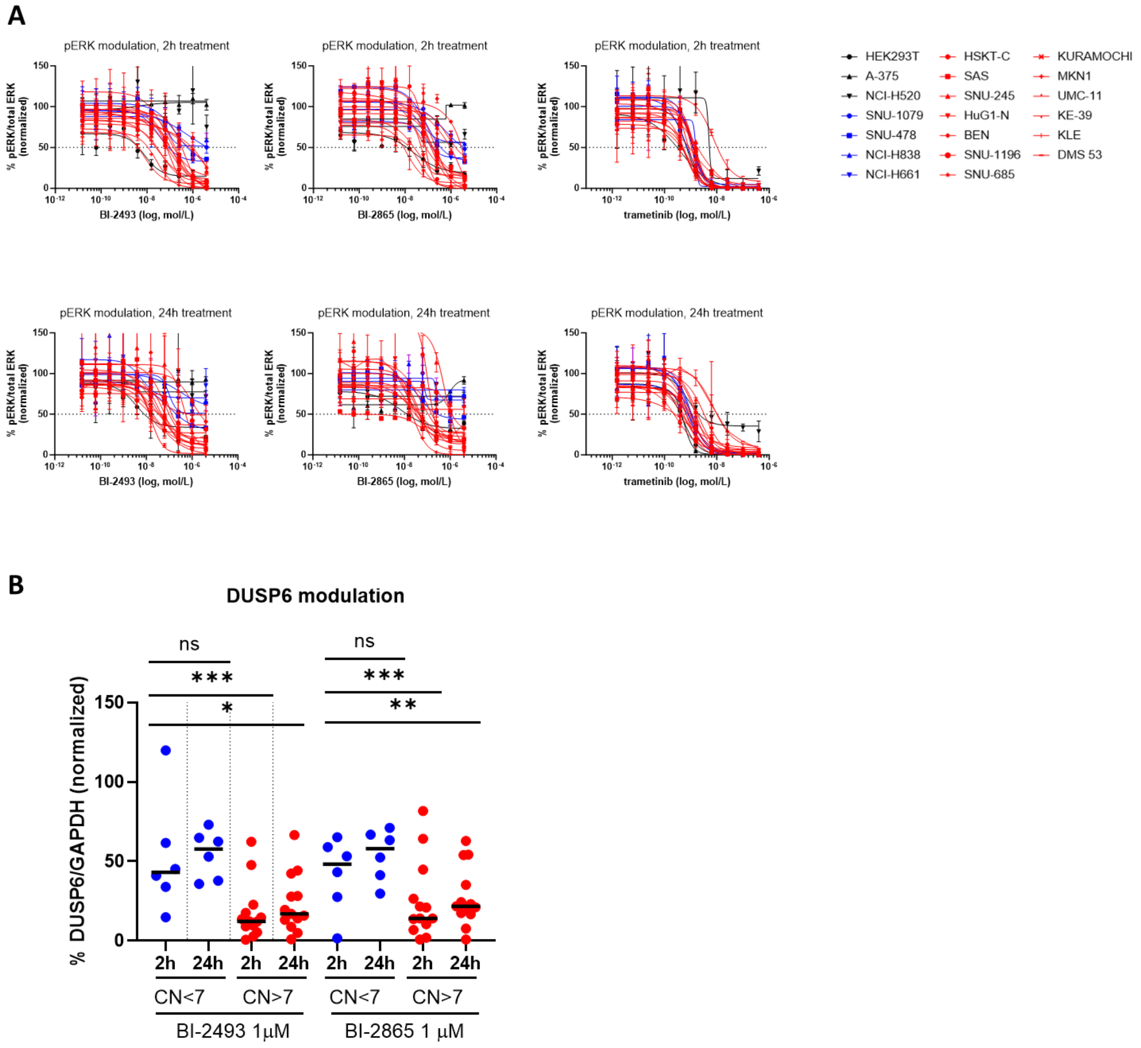

Supplementary Figure 8: *KRAS wild-type amplified cancer cell lines are sensitive to pan-KRAS inhibitors BI-2493 and BI-2865.* **(A)** Inhibition of pERK by BI-2493, BI-2865 and trametinib at the indicated timepoints and for the indicated cell lines (n=2, means± SD). Control cell lines, cell lines with KRAS wild-type CN<7 and cell lines with KRAS wild-type CN>7 are colored in black, blue, and red, respectively. (**B**) Quantification of down regulation of pERK from (A) at 1μM concentration of BI-2493 and BI-2865. P-values were calculated using two-way ANOVA, followed by Tukey's multiple comparisons test.

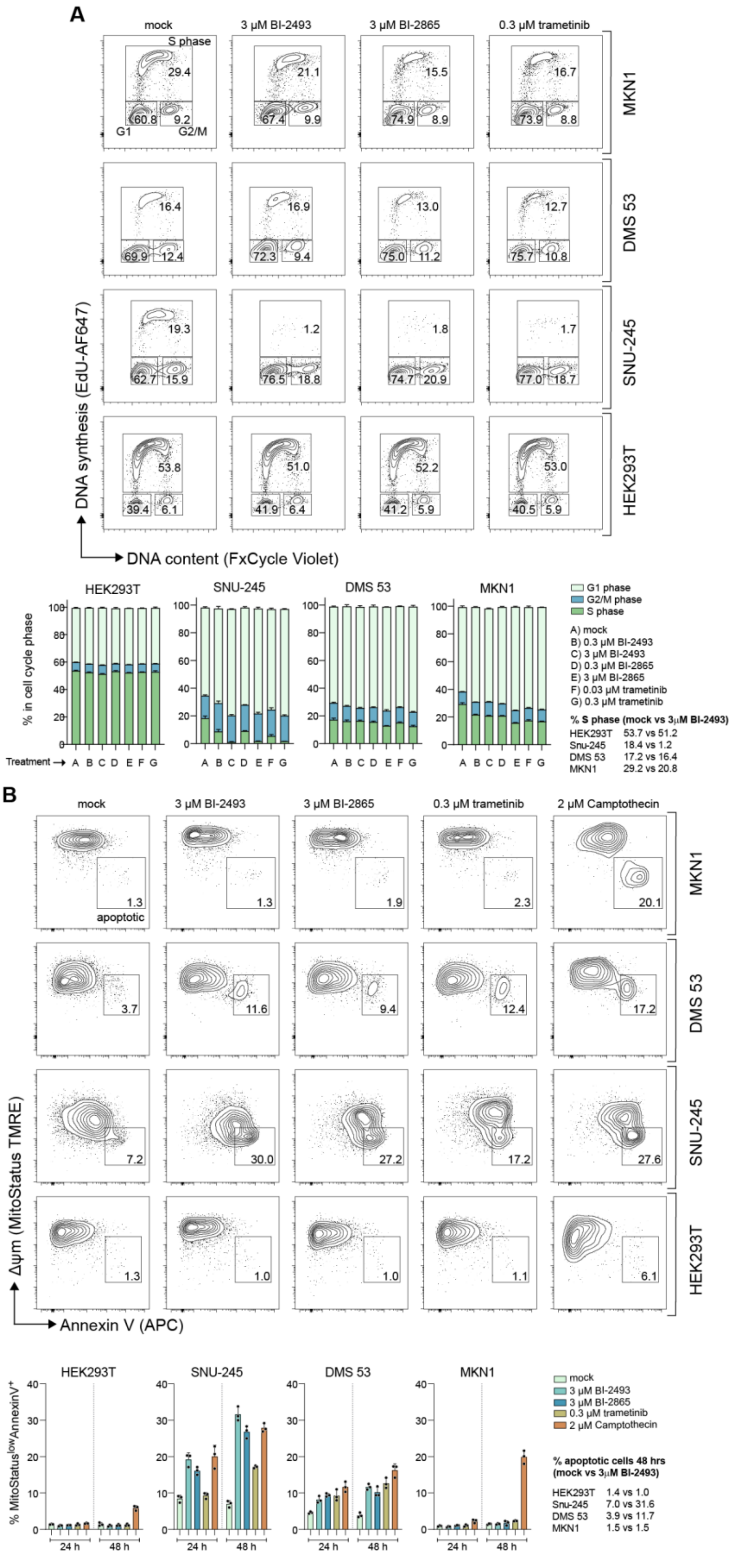
A
mock
3 µM BI-2493
3 µM BI-2865
0.3 µM trametinib
MKN1
DMS 53
SNU-245
HEK293T
DNA synthesis (EdU-AF647)
DNA content (FxCycle Violet)
% in cell cycle phase
G1 phase
G2/M phase
S phase
A) mock
B) 0.3 µM BI-2493
C) 3 µM BI-2493
D) 0.3 µM BI-2865
E) 3 µM BI-2865
F) 0.03 µM trametinib
G) 0.3 µM trametinib
% S phase (mock vs 3µM BI-2493)
HEK293T 53.7 vs 51.2
Snu-245 18.4 vs 1.2
DMS 53 17.2 vs 16.4
MKN1 29.2 vs 20.8
Treatment
B
2 µM Camptothecin
apoptotic
Δψm (MitoStatus TMRE)
Annexin V (APC)
% MitoStatus^low AnnexinV^+
24 h
48 h
mock
3 µM BI-2493
3 µM BI-2865
0.3 µM trametinib
2 µM Camptothecin
% apoptotic cells 48 hrs (mock vs 3µM BI-2493)
HEK293T 1.4 vs 1.0
Snu-245 7.0 vs 31.6
DMS 53 3.9 vs 11.7
MKN1 1.5 vs 1.5

Supplementary Figure 9: *BI-2493 and BI-2865 treatment induces cell cycle arrest and apoptosis in KRAS wild-type amplified cancer cell lines*. **(A)** Upper panel: Representative flow blots of cell cycle states determined by EdU incorporation into newly synthesized DNA and total DNA content staining by FxCycle of indicated cell lines treated for 48 h with DMSO (mock), 3 µM BI-2493, 3 µM BI-2865 and 0.3 µM trametinib. Cells were pre-gated based on scattering properties and DNA content. Numbers indicate frequency of parent population. Lower panel: Impact of 48 h treatment with DMSO (mock), BI-2493, BI-2865 or trametinib at the indicated concentrations on cell cycle states of the indicated cell lines (N=3 (assay was run in triplicates), means + SD) **(B)** Upper panel: Representative flow blots of induction of apoptosis after 48 h of treatment of the indicated cell lines with DMSO (mock), 3 µM BI-2493, 3 µM BI-2865, 0.3 µM trametinib and 2 µM Camptothecin. Cells were pre-gated based on their scattering properties. Apoptotic cells were defined by loss of inner mitochondrial membrane potential ($\Delta\psi m$) and detection of phosphatidylserine by Annexin V staining. Numbers indicate frequency of parent population. Lower panel: Induction of apoptosis in the indicated cell lines by treatment with DMSO (mock), BI-2493, BI-2865, trametinib or Camptothecin at the indicated concentrations after 24 and 48 h determined by flow cytometry (N=3 (assay was run in triplicates), means + SD).

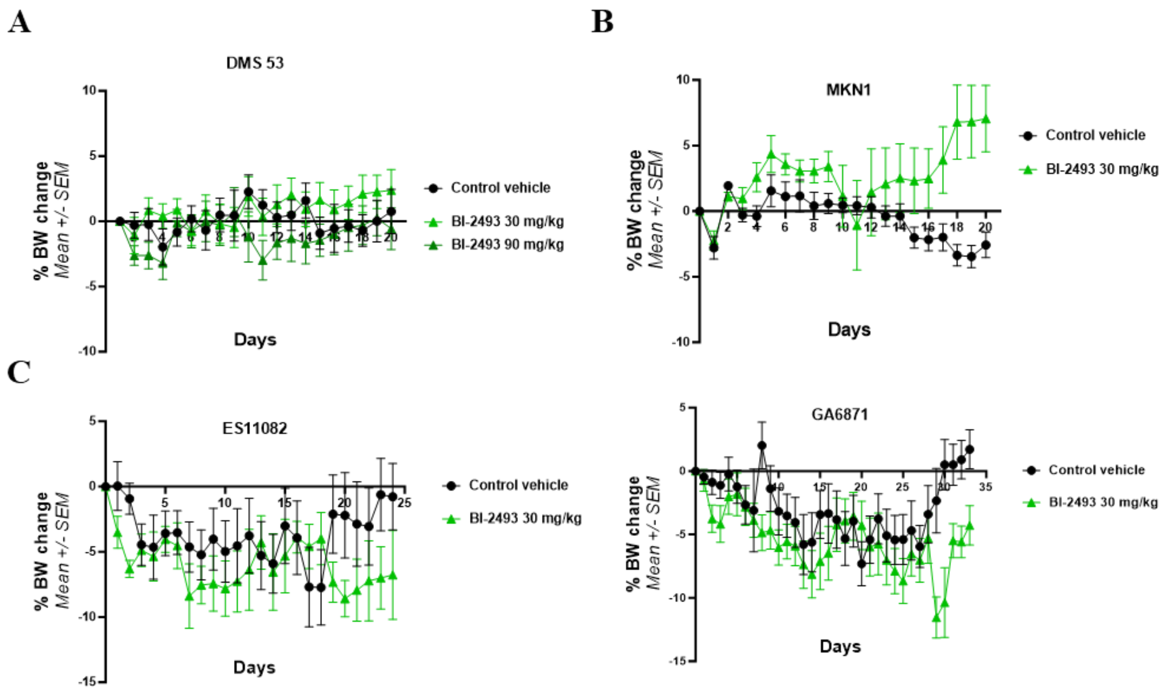

Supplementary Figure 10. *BI-2493 treatment in animal models is tolerated.* % bodyweight change in xenograft models treated with control vehicle or BI-2493. Data represent the mean % bodyweight change +/- SEM of mice grafted with: (**A**) DMS 53 cells (N = 7); (**B**) MKN1 cells (N = 7). One animal in the BI-2493 treated group had to be sacrificed earlier (d11) due to bodyweight loss. (**C**) ES11082 PDX model (N = 8). Two animals in the control vehicle treated group and three animals in the BI-2493 treated group had to be sacrificed earlier (d18, d18, d15, d12, d7, respectively). (**D**) GA6871 PDX model (N = 8). One animal in the control vehicle treated group and 2 animals in the BI-2493 treated group had to be sacrificed earlier (d7 and d16, d30, respectively) due to bodyweight loss.

References

1. East, P. *et al.* RAS oncogenic activity predicts response to chemotherapy and outcome in lung adenocarcinoma. *Nat. Commun.* **13**, 5632 (2022).

2. Holderfield, M. *et al.* Concurrent inhibition of oncogenic and wild-type RAS-GTP for cancer therapy. *Nature* 1–8 (2024) doi:10.1038/s41586-024-07205-6.

3. Wagle, M.-C. *et al.* A transcriptional MAPK Pathway Activity Score (MPAS) is a clinically relevant biomarker in multiple cancer types. *npj Precis. Oncol.* **2**, 7 (2018).
