# Supplementary material for: Pan-KRAS Inhibitors BI-2493 and BI-2865 Display Potent Antitumor Activity in Tumors with KRAS Wild-type Allele Amplification
Source: Mol Cancer Ther. 2024 Dec 21;24(4):550–62. doi: 10.1158/1535-7163.MCT-24-0386 (PMC11962398; doi:10.1158/1535-7163.MCT-24-0386)
Supplement: Supplementary Figure 5 — Anti-proliferative activity of RMC-7977 across different KRAS altered cell lines. (Left) Anti-proliferative activity of RMC-7977 (2) across different KRAS mutant or KRAS wild-type amplified cell lines. Cell lines are sorted by median sensitivity across KRAS alleles. Note: AUC values are relative measures of drug sensitivity and are therefore suitable to compare drug sensitivity across cell lines for a single compound but do not allow for a comparison across compounds. (Right) Comparison of sensitivity values for RMC-7977 (2) for cell lines with dependency on either KRAS, HRAS or NRAS. Cell lines with a Chronos score (gene effect score) of less than -1 were considered dependent. Sensitivity means between groups were tested for significance using a one-sided Wilcoxon-test. [file mct-24-0386_supplementary_figure_5_supps5.pdf]

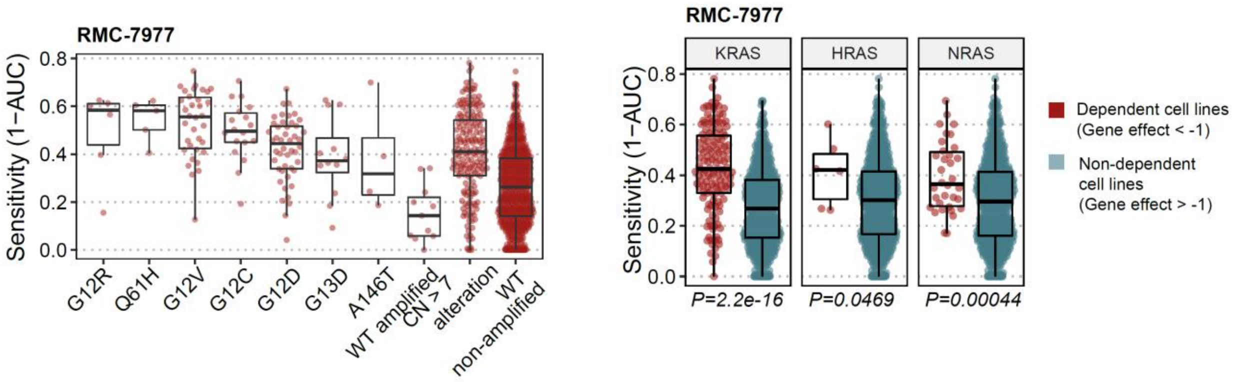

Supplementary Figure 5. *Anti-proliferative activity of RMC-7977 across different KRAS altered cell lines.* **(Left)** Anti-proliferative activity of RMC-7977 (Ref. 40) across different KRAS mutant or KRAS wild-type amplified cell lines. Cell lines are sorted by median sensitivity across KRAS alleles. Note: AUC values are relative measures of drug sensitivity and are therefore suitable to compare drug sensitivity across cell lines for a single compound but do not allow for a comparison across compounds. **(Right)** Comparison of sensitivity values for RMC-7977 (Ref. 40) for cell lines with dependency on either KRAS, HRAS or NRAS. Cell lines with a Chronos score (gene effect score) of less than -1 were considered dependent. Sensitivity means between groups were tested for significance using a one-sided Wilcoxon-test.
