# Supplementary material for: Pan-KRAS Inhibitors BI-2493 and BI-2865 Display Potent Antitumor Activity in Tumors with KRAS Wild-type Allele Amplification
Source: Mol Cancer Ther. 2024 Dec 21;24(4):550–62. doi: 10.1158/1535-7163.MCT-24-0386 (PMC11962398; doi:10.1158/1535-7163.MCT-24-0386)
Supplement: Supplementary Figure 6 — Relationship between KRAS wild-type amplification and KRAS oncogenic activity in TCGA patient data. RAS activation signatures MPAS (3), RAS_addiction and Ras84 (1). Enrichment scores were estimated using single sample enrichment (ssGSEA) in TCGA patient data. A one-sided Wilcox-test was used to test for significance between KRAS relative copy number of 2-7 or >7. [file mct-24-0386_supplementary_figure_6_supps6.pdf]

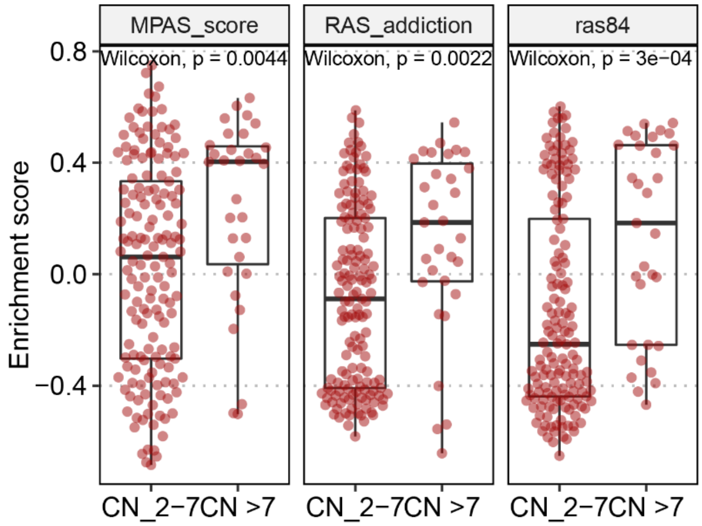

Supplementary Figure 6: *Relationship between KRAS wild-type amplification and KRAS oncogenic activity in TCGA patient data*. RAS activation signatures MPAS (Ref. 37), RAS_addiction and Ras84 (Ref 38). Enrichment scores were estimated using single sample enrichment (ssGSEA) in TCGA patient data. A one-sided Wilcox-test was used to test for significance between KRAS relative copy number of 2-7 or >7.
