# Supplementary material for: Pan-KRAS Inhibitors BI-2493 and BI-2865 Display Potent Antitumor Activity in Tumors with KRAS Wild-type Allele Amplification
Source: Mol Cancer Ther. 2024 Dec 21;24(4):550–62. doi: 10.1158/1535-7163.MCT-24-0386 (PMC11962398; doi:10.1158/1535-7163.MCT-24-0386)
Supplement: Supplementary Figure 7 — Gastroesophageal cancers are enriched in KRAS wild-typeWT amplified tumors. Gastroesophageal cancers (n=3464) were selected from the AACR Genie MSK and DFCI cohort (version v16.0-public). Only patients with any alterations in the above listed genes are shown (1498 unaltered patients are not shown). Co-alterations are ranked by frequency. KRAS amplified samples are defined with a GISTIC score of 2 according to AACR GENIE as exact copy number thresholds are not available from the AACR GENIE cohort. [file mct-24-0386_supplementary_figure_7_supps7.pdf]

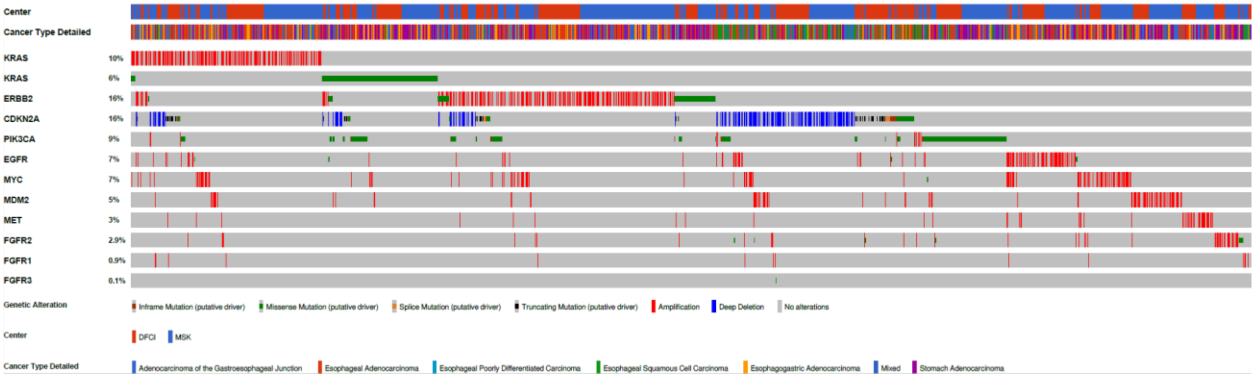

Supplementary Figure 7. *Gastroesophageal cancers are enriched in KRAS wild-type amplified tumors*. Gastroesophageal cancers *(n=3464)* were selected from the AACR Genie MSK and DFCI cohort (version v16.0-public). Only patients with any alterations in the above listed genes are shown (1498 unaltered patients are not shown). Co-alterations are ranked by frequency. KRAS amplified samples are defined with a GISTIC score of 2 according to AACR GENIE as exact copy number thresholds are not available from the AACR GENIE cohort.
