# Supplementary material for: Pan-KRAS Inhibitors BI-2493 and BI-2865 Display Potent Antitumor Activity in Tumors with KRAS Wild-type Allele Amplification
Source: Mol Cancer Ther. 2024 Dec 21;24(4):550–62. doi: 10.1158/1535-7163.MCT-24-0386 (PMC11962398; doi:10.1158/1535-7163.MCT-24-0386)
Supplement: Supplementary Figure 8 — KRAS wild-type amplified cancer cell lines are sensitive to pan-KRAS inhibitors BI-2493 and BI-2865. (A) Inhibition of pERK by BI-2493, BI-2865 and trametinib at the indicated timepoints and for the indicated cell lines (n=2, means ± SD). Control cell lines, cell lines with KRAS wild-type CN<7 and cell lines with KRAS wild-type CN>7 are colored in black, blue, and red, respectively. (B) Quantification of down regulation of pERK from (A) at 1M concentration of BI-2493 and BI-2865. P-values were calculated using two-way ANOVA, followed by Tukey’s multiple comparisons test. [file mct-24-0386_supplementary_figure_8_supps8.pdf]

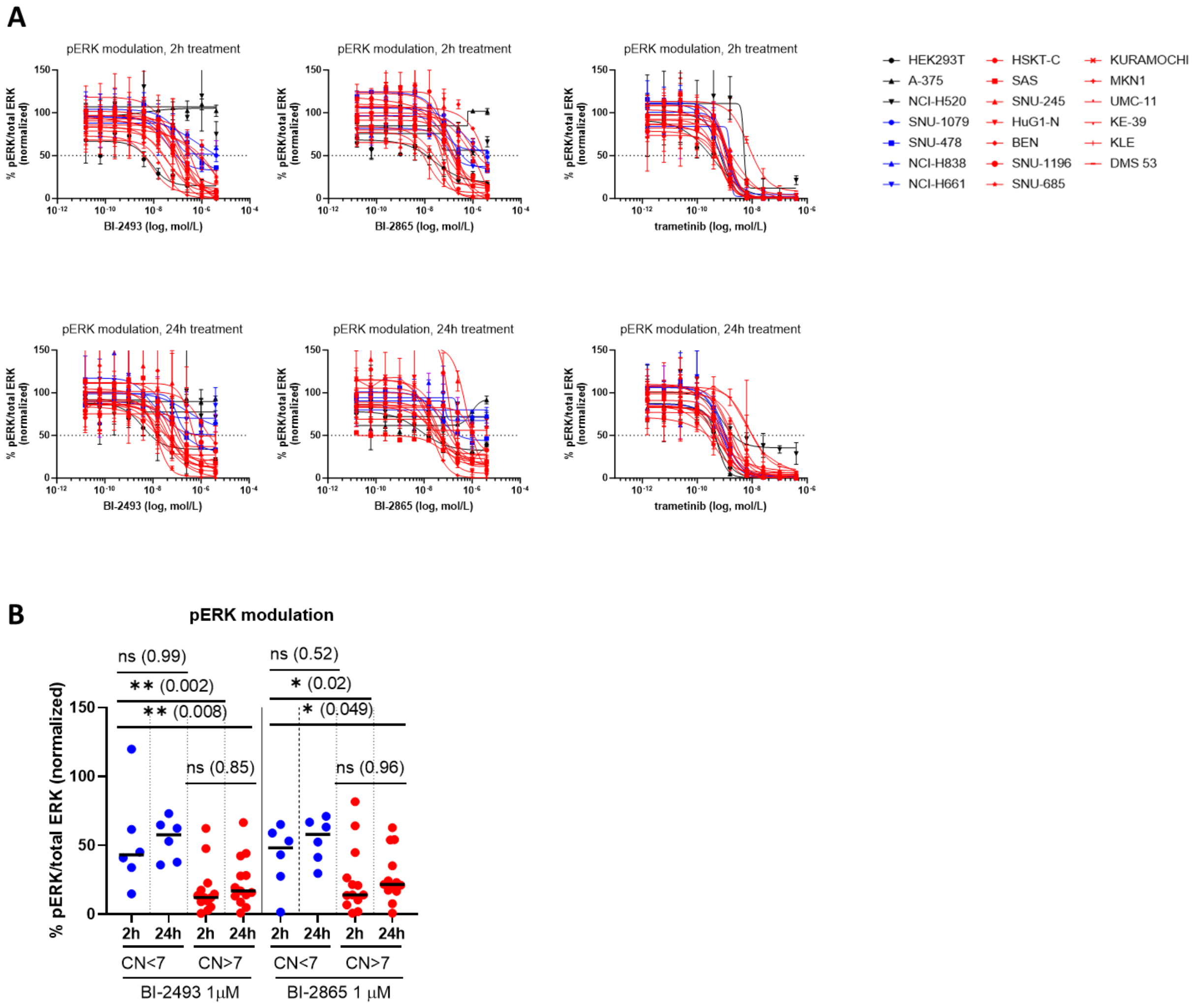

Supplementary Figure 8: *KRAS wild-type amplified cancer cell lines are sensitive to pan-KRAS inhibitors BI-2493 and BI-2865.* **(A)** Inhibition of pERK by BI-2493, BI-2865 and trametinib at the indicated timepoints and for the indicated cell lines (n=2, means± SD). Control cell lines, cell lines with KRAS wild-type CN<7 and cell lines with KRAS wild-type CN>7 are colored in black, blue, and red, respectively. (**B**) Quantification of down regulation of pERK from (A) at 1µM concentration of BI-2493 and BI-2865. P-values were calculated using two-way ANOVA, followed by Tukey's multiple comparisons test.
