# Supplementary material for: Pan-KRAS Inhibitors BI-2493 and BI-2865 Display Potent Antitumor Activity in Tumors with KRAS Wild-type Allele Amplification
Source: Mol Cancer Ther. 2024 Dec 21;24(4):550–62. doi: 10.1158/1535-7163.MCT-24-0386 (PMC11962398; doi:10.1158/1535-7163.MCT-24-0386)
Supplement: Supplementary Figure 9 — BI-2493 and BI-2865 treatment induces cell cycle arrest and apoptosis in KRAS wild-type amplified cancer cell lines. (A) Upper panel: Representative flow blots of cell cycle states determined by EdU incorporation into newly synthesized DNA and total DNA content staining by FxCycle of indicated cell lines treated for 48 h with DMSO (mock), 3 µM BI-2493, 3 µM BI-2865 and 0.3 µM trametinib. Cells were pre-gated based on scattering properties and DNA content. Numbers indicate frequency of parent population. Lower panel: Impact of 48 h treatment with DMSO (mock), BI-2493, BI-2865 or trametinib at the indicated concentrations on cell cycle states of the indicated cell lines (N=3 (assay was run in triplicates), means + SD) (B) Upper panel: Representative flow blots of induction of apoptosis after 48 h of treatment of the indicated cell lines with DMSO (mock), 3 µM BI-2493, 3 µM BI-2865, 0.3 µM trametinib and 2 µM Camptothecin. Cells were pre-gated based on their scattering properties. Apoptotic cells were defined by loss of inner mitochondrial membrane potential (Δψm) and detection of phosphatidylserine by Annexin V staining. Numbers indicate frequency of parent population. Lower panel: Induction of apoptosis in the indicated cell lines by treatment with DMSO (mock), BI-2493, BI-2865, trametinib or Camptothecin at the indicated concentrations after 24 and 48 h determined by flow cytometry (N=3 (assay was run in triplicates), means +SD). [file mct-24-0386_supplementary_figure_9_supps9.pdf]

A

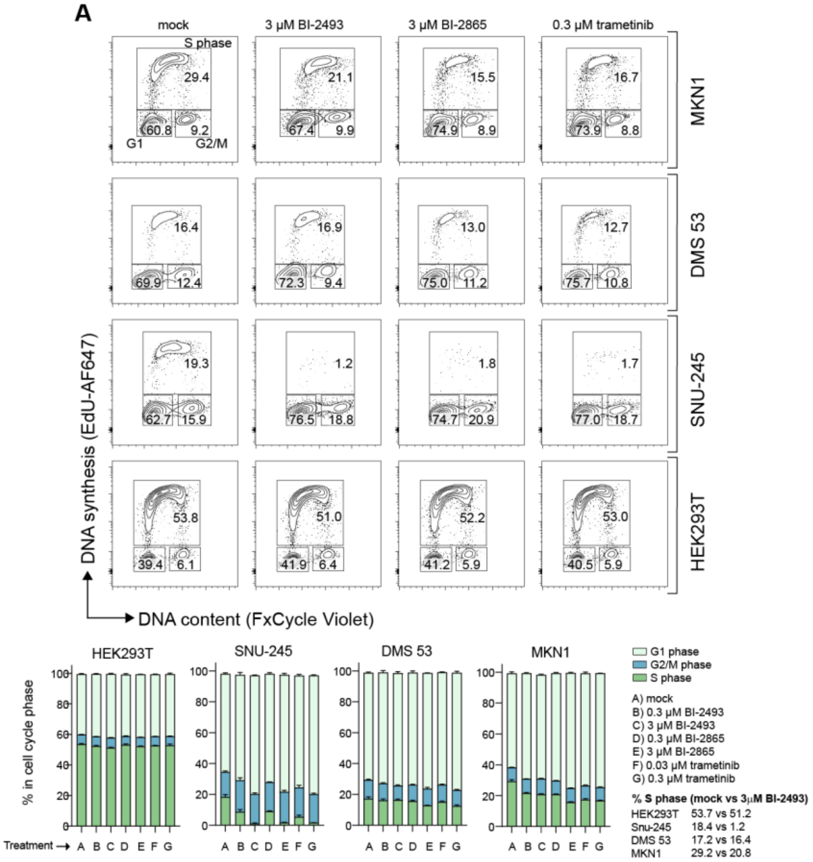

mock
3 μM BI-2493
3 μM BI-2865
0.3 μM trametinib
S phase
G1
G2/M
MKN1
DMS 53
SNU-245
HEK293T
DNA synthesis (EdU-AF647)
DNA content (FxCycle Violet)
29.4
60.8
9.2
21.1
67.4
9.9
15.5
74.9
8.9
16.7
73.9
8.8
16.4
69.9
12.4
16.9
72.3
9.4
13.0
75.0
11.2
12.7
75.7
10.8
19.3
62.7
15.9
1.2
76.5
18.8
1.8
74.7
20.9
1.7
77.0
18.7
53.8
39.4
6.1
51.0
41.9
6.4
52.2
41.2
5.9
53.0
40.5
5.9
% in cell cycle phase
Treatment →
A B C D E F G
G1 phase
G2/M phase
S phase
A) mock
B) 0.3 μM BI-2493
C) 3 μM BI-2493
D) 0.3 μM BI-2865
E) 3 μM BI-2865
F) 0.03 μM trametinib
G) 0.3 μM trametinib
% S phase (mock vs 3μM BI-2493)
HEK293T 53.7 vs 51.2
Snu-245 18.4 vs 1.2
DMS 53 17.2 vs 16.4
MKN1 29.2 vs 20.8

B

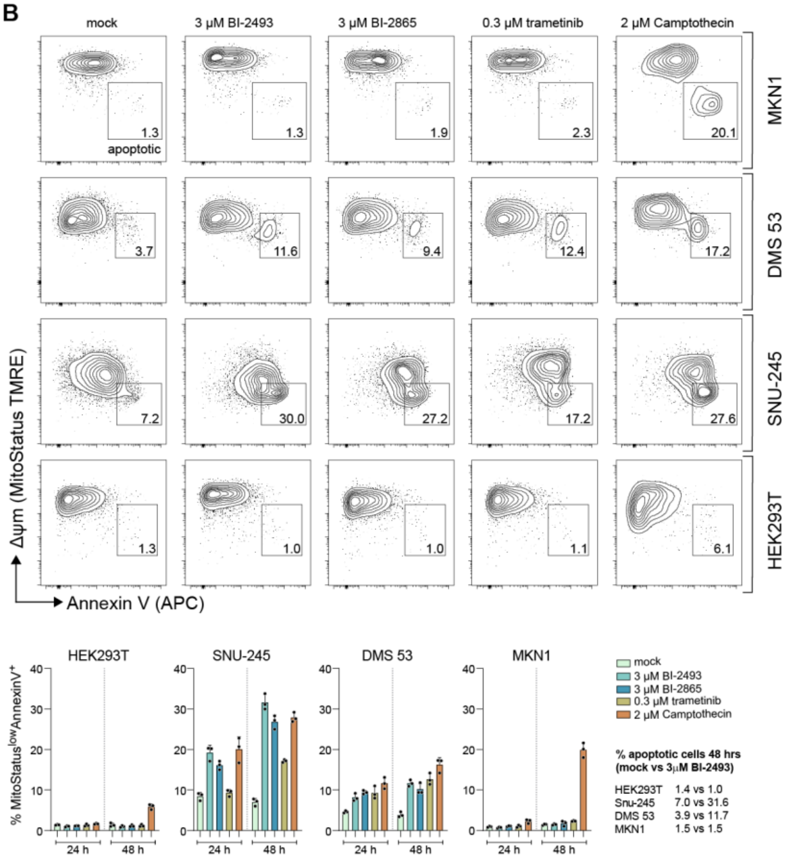

mock
3 μM BI-2493
3 μM BI-2865
0.3 μM trametinib
2 μM Camptothecin
apoptotic
MKN1
DMS 53
SNU-245
HEK293T
Δψm (MitoStatus TMRE)
Annexin V (APC)
1.3
1.3
1.9
2.3
20.1
3.7
11.6
9.4
12.4
17.2
7.2
30.0
27.2
17.2
27.6
1.3
1.0
1.0
1.1
6.1
% MitoStatus^low AnnexinV^+
24 h
48 h
mock
3 μM BI-2493
3 μM BI-2865
0.3 μM trametinib
2 μM Camptothecin
% apoptotic cells 48 hrs (mock vs 3μM BI-2493)
HEK293T 1.4 vs 1.0
Snu-245 7.0 vs 31.6
DMS 53 3.9 vs 11.7
MKN1 1.5 vs 1.5

Supplementary Figure 9: *BI-2493 and BI-2865 treatment induces cell cycle arrest and apoptosis in KRAS wild-type amplified cancer cell lines*. **(A)** Upper panel: Representative flow blots of cell cycle states determined by EdU incorporation into newly synthesized DNA and total DNA content staining by FxCycle of indicated cell lines treated for 48 h with DMSO (mock), 3 µM BI-2493, 3 µM BI-2865 and 0.3 µM trametinib. Cells were pre-gated based on scattering properties and DNA content. Numbers indicate frequency of parent population. Lower panel: Impact of 48 h treatment with DMSO (mock), BI-2493, BI-2865 or trametinib at the indicated concentrations on cell cycle states of the indicated cell lines (N=3 (assay was run in triplicates), means + SD) **(B)** Upper panel: Representative flow blots of induction of apoptosis after 48 h of treatment of the indicated cell lines with DMSO (mock), 3 µM BI-2493, 3 µM BI-2865, 0.3 µM trametinib and 2 µM Camptothecin. Cells were pre-gated based on their scattering properties. Apoptotic cells were defined by loss of inner mitochondrial membrane potential (Δψm) and detection of phosphatidylserine by Annexin V staining. Numbers indicate frequency of parent population. Lower panel: Induction of apoptosis in the indicated cell lines by treatment with DMSO (mock), BI-2493, BI-2865, trametinib or Camptothecin at the indicated concentrations after 24 and 48 h determined by flow cytometry (N=3 (assay was run in triplicates), means + SD).
