# Supplementary material for: Pan-KRAS Inhibitors BI-2493 and BI-2865 Display Potent Antitumor Activity in Tumors with KRAS Wild-type Allele Amplification
Source: Mol Cancer Ther. 2024 Dec 21;24(4):550–62. doi: 10.1158/1535-7163.MCT-24-0386 (PMC11962398; doi:10.1158/1535-7163.MCT-24-0386)
Supplement: Supplementary Figure 10 — BI-2493 treatment in animal models is tolerated. % bodyweight change in xenograft models treated with control vehicle or BI-2493. Data represent the mean % bodyweight change +/- SEM of mice grafted with: (A) DMS 53 cells (N=7); (B) MKN1 cells (N=7). One animal in the BI-2493 treated group had to be sacrificed earlier (d11) due to bodyweight loss. (C) ES11082 PDX model (N=8). Two animals in the control vehicle treated group and three animals in the BI-2493 treated group had to be sacrificed earlier (d18, d18, d15, d12, d7, respectively). (D) GA6871 PDX model (N=8). One animal in the control vehicle treated group and 2 animals in the BI-2493 treated group had to be sacrificed earlier (d7 and d16, d30, respectively) due to bodyweight loss. [file mct-24-0386_supplementary_figure_10_supps10.pdf]

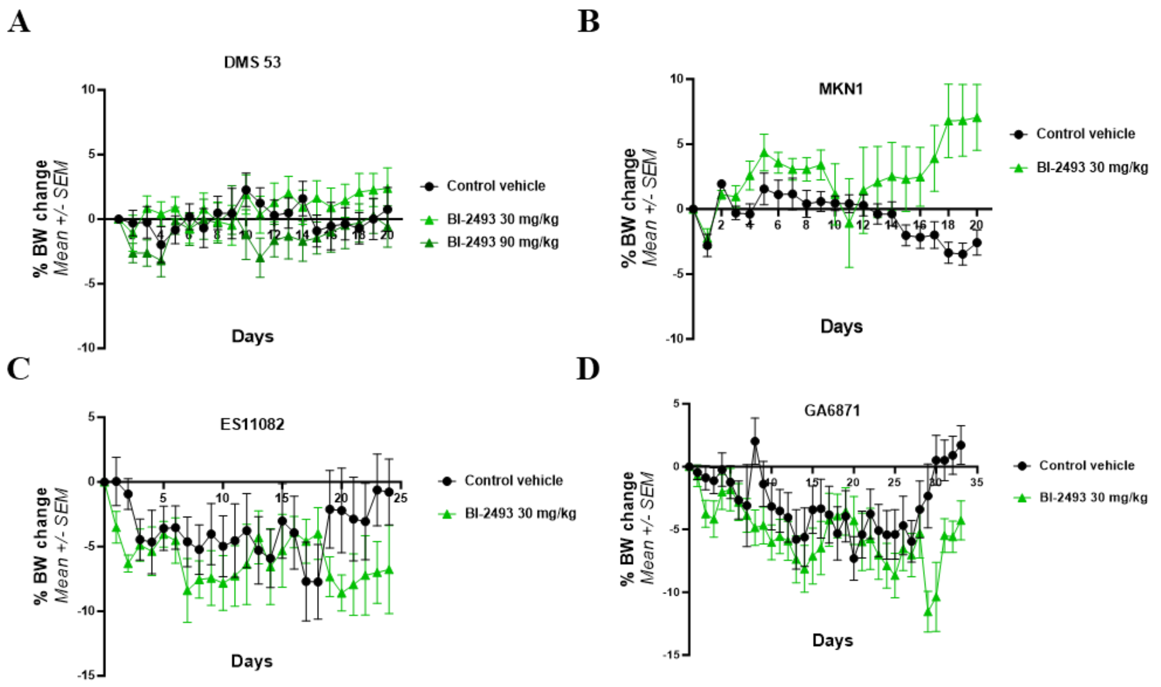

Supplementary Figure 10. *BI-2493 treatment in animal models is tolerated.* % bodyweight change in xenograft models treated with control vehicle or BI-2493. Data represent the mean % bodyweight change +/- SEM of mice grafted with: (**A**) DMS 53 cells (N = 7); (**B**) MKN1 cells (N = 7). One animal in the BI-2493 treated group had to be sacrificed earlier (d11) due to bodyweight loss. (**C**) ES11082 PDX model (N = 8). Two animals in the control vehicle treated group and three animals in the BI-2493 treated group had to be sacrificed earlier (d18, d18, d15, d12, d7, respectively). (**D**) GA6871 PDX model (N = 8). One animal in the control vehicle treated group and 2 animals in the BI-2493 treated group had to be sacrificed earlier (d7 and d16, d30, respectively) due to bodyweight loss.
